# Rational A‐Site Entropy Engineering in Perovskites: Dual‐Exchange Enhanced Magnetoelectric Coupling for Ultra‐Efficient Microwave Absorption

**DOI:** 10.1002/advs.202516938

**Published:** 2025-10-17

**Authors:** Mengru Li, Kaiyue Zhao, Bingbing Fan, Yang Li, Dalong Tan, Hailong Wang, Qilong Gao, Wei Li, Hongsong Zhang, Yanqiu Zhu, Rui Zhang

**Affiliations:** ^1^ School of Materials Science and Engineering Zhengzhou University Zhengzhou 450001 China; ^2^ School of Physics Zhengzhou University Zhengzhou 450001 China; ^3^ Henan Academy of Sciences Zhengzhou 450046 China; ^4^ Department of Engineering Faculty of Environment Science and Economy University of Exeter Exeter EX4 4QF UK; ^5^ State Key Laboratory of Structural Analysis Optimization and CAE Software for Industrial Equipment National Engineering Research Center for Advanced Polymer Processing Technology Zhengzhou University Zhengzhou 450002 China; ^6^ Mechanical Engineering Henan University of Engineering Zhengzhou 451151 China; ^7^ School of Materials Science and Engineering Luoyang Institute of Technology Luoyang 471000 China

**Keywords:** electromagnetic absorption, high‐entropy, magnetism, perovskites

## Abstract

High‐entropy engineering at the A‐site, combined with the variable valence states of Mn ions and diverse bonding configurations of perovskite elements and structures, presents new opportunities for the development and application of high‐temperature electromagnetic wave‐absorbing materials. In this study, the magnetic and dielectric properties of AMnO_3_ are controlled by designing A‐site elements with various ionic radii and entropies. The microwave‐absorption performance of (La_0.2_Ba_0.2_Sr_0.2_Ca_0.2_Na_0.2_)MnO_3_ high‐entropy perovskites is significantly higher than those of AMnO_3_ with different ionic radii and (Ba_1/3_Sr_1/3_Ca_1/3_)MnO_3_ medium‐entropy perovskites. Specifically, the high‐entropy samples exhibit a minimum reflection loss (RL_min_) of −60.86 dB at a thickness of 1.0 mm and an effective absorption bandwidth of 3.26 GHz, whereas the medium‐entropy ceramics show RL_min_ values of −17.93 and −44.59 dB at 8.5 mm ((Ba_1/3_Sr_1/3_Ca_1/3_)MnO_3_) and 8.8 mm ((La_0.25_Ba_0.25_Sr_0.25_Ca_0.25_)MnO_3_), respectively. In high‐entropy perovskites, aliovalent ions and oxygen vacancies at the A‐site promote exchange interactions between Mn─O─Mn bonds, enhancing magnetism. Additionally, oxygen vacancies and lattice distortions in high‐entropy systems enhance the dielectric loss, achieving magnetoelectric cooperative coupling in high‐entropy perovskites. This work provides a new research direction for designing single‐phase perovskites with excellent electromagnetic wave‐absorbing properties via magnetoelectric cooperative‐loss coupling.

## Graphical abstract

Rational A‐site entropy engineering in perovskites induces dual‐exchange‐enhanced magnetoelectric coupling, achieving ultra‐efficient microwave absorption with superior reflection loss and broad bandwidth.

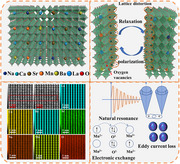

## 1 Introduction

Supersonic aircraft hold significant importance in military strategy, with surface temperatures of critical components, such as the nose cone and engine, reaching 1000 °C or higher. As a result, the external thermal‐protection layer must provide stealth capabilities and offer high‐temperature resistance.^[^ ^1^ ^]^ Beyond this point, the polymer matrices decompose, and magnetic metals exhibit a significant reduction in magnetic‐loss capabilities owing to their Curie temperatures. Therefore, the performance of ceramics has received increasing attention.^[^ ^2^, ^3^ ^]^

Perovskites (ABO_3_) are compounds that contain various cations and oxygen ions.^[^ ^4^, ^5^ ^]^ B‐site cations, typically transition‐metal ions with smaller radii, occupy the octahedral gaps formed by oxygen ions, creating a BO_6_ octahedral structure. A‐site cations, which are usually larger alkali or alkaline‐earth metal ions and lanthanide rare‐earth ions, occupy the spaces between these octahedra. The radius difference between the A‐ and B‐sites influences the torsion and deformation of the octahedra and their connections, resulting in structurally diverse perovskites, such as tetragonal, cubic,^[^ ^6^ ^]^ hexagonal,^[^ ^7^ ^]^ and double perovskites.^[^ ^8^, ^9^ ^]^ Their structural and elemental diversity endows perovskites with a wealth of physical and chemical properties, ranging from ferromagnetism to antiferromagnetism,^[^ ^10^ ^]^ giant magnetoresistance,^[^ ^11^ ^]^ and high dielectric properties.^[^ ^12^, ^13^ ^]^

High‐entropy ceramics, which are characterized by high entropy, severe lattice distortion, sluggish diffusion, and cocktail effects, provide additional application possibilities. These ceramics can accommodate elements with large radial discrepancies at the same lattice site, forming a consolidated single‐crystal structure that transcends the structural barriers of various perovskite types.^[^ ^14^, ^15^ ^]^ The properties of these materials are determined by their compositional complexity and local interactions, suggesting that the high‐entropy theory can effectively regulate the absorption properties of materials.^[^ ^16^ ^]^ The electronic structures and lattices of the obtained samples can be tailored to achieve the desired properties by utilizing short‐range chemical disorder.^[^ ^17^ ^]^ For example, Zhao et al. reported that adjusting the entropy configuration produced obvious grain boundaries, oxygen defects, and ultradense distorted lattices, leading to improved attenuation performance through strong interface and defect polarization.^[^ ^18^ ^]^ Ma et al. revealed that the magnetic properties of high‐entropy ceramics are closely related to the superexchange interactions of magnetic iron‐group elements between tetrahedral and octahedral ions and the arrangement of electrons in different orbitals.^[^ ^19^ ^]^ Dai demonstrated that the formation of numerous oxygen vacancies in two‐phase entropy materials accelerates electron or ion migration, thereby enhancing dielectric‐loss capacity.^[^ ^20^ ^]^ The biphase structure promotes interfacial polarization, while the abundance of oxygen vacancies increases dipole polarization. In addition, the introduction of magnetic elements induces magnetic loss to electromagnetic waves, resulting in a synergistic effect of dielectric and magnetic loss. Therefore, using a high‐entropy design for element selection and substitution to adjust the perovskite structure and optimize a single dielectric‐loss mechanism is highly feasible.^[^ ^21^, ^22^ ^]^

Perovskite manganese oxides (AMnO_3_) exhibit various exotic ordering phenomena, including charge, orbital, and spin ordering. Mn atoms with variable valence states (d^5^4s^2^) provide adjustable chemical states and magnetic properties within the ABO_3_ structure.^[^ ^23^ ^]^ In our previous work, the introduction of a high‐entropy design at the B‐site induced distortions in the oxygen vacancies and octahedral structures of perovskite materials. These structural changes enabled the precise tuning of the dielectric and magnetic properties, significantly enhancing the electromagnetic wave‐absorption performance of the material.^[^ ^24^ ^]^ However, the high‐entropy design at the B‐site can negatively affect the magnetic properties of perovskites.

When the A‐site is occupied by alkaline earth metals (e.g., Ca, Sr, and Ba), the structure features a high oxidation state of Mn^4+^. Consequently, AMnO_3_ (*A* = Ca, Sr, Ba) exhibits significant differences in crystal structure and performance as the radius of the alkali earth‐metal ions changes. For example, CaMnO_3_, an orthorhombic system, is known for its strong insulation properties, giant magnetoresistance effect,^[^ ^25^ ^]^ and thermoelectric effects.^[^ ^26^, ^27^ ^]^ Substituting Sr^2+^ or Ba^2+^ for Ca^2+^ increases the ionic radii of the A‐site and Mn, distorting and rearranging the MnO_6_ octahedra into a coplanar or arch configuration, resulting in a hexagonal crystal structure (e.g., SrMnO_3_ 4H, BaMnO_3_ 2H).^[^ ^28^ ^]^ Doping with rare‐earth ions further alters the structure and properties of the perovskite, affecting its conductivity and magnetic properties through changes in the valence states of adjacent Mn ions.^[^ ^29^, ^30^ ^]^ Therefore, high‐entropy engineering at the A‐site, combined with the variable valence states of Mn ions and diverse bonding configurations of perovskite elements and structures, may present new opportunities for the development and application of electromagnetic wave‐absorbing materials.

To verify this hypothesis, entropy engineering is applied at the A‐site to improve the dielectric performance of AMnO_3_ while maintaining its magnetic properties, thereby achieving superior microwave‐absorption capabilities. This study regulates the magnetic and dielectric properties of AMnO_3_ by designing A‐site elements with different radii or entropy. The influence of the species, magnitude, and valence state of the A‐site cations on the octahedral structure of MnO_6_ and the exchange between electrons are investigated with the aim of regulating the perovskite structure and enhancing the absorption performance. Efficient electromagnetic‐wave absorption is achieved through the synergistic effect of dielectric and magnetic losses facilitated by the high‐entropy design.

## 2 Results and Discussion

### 2.1 Crystal and Structure Analysis

**Figure 1:**
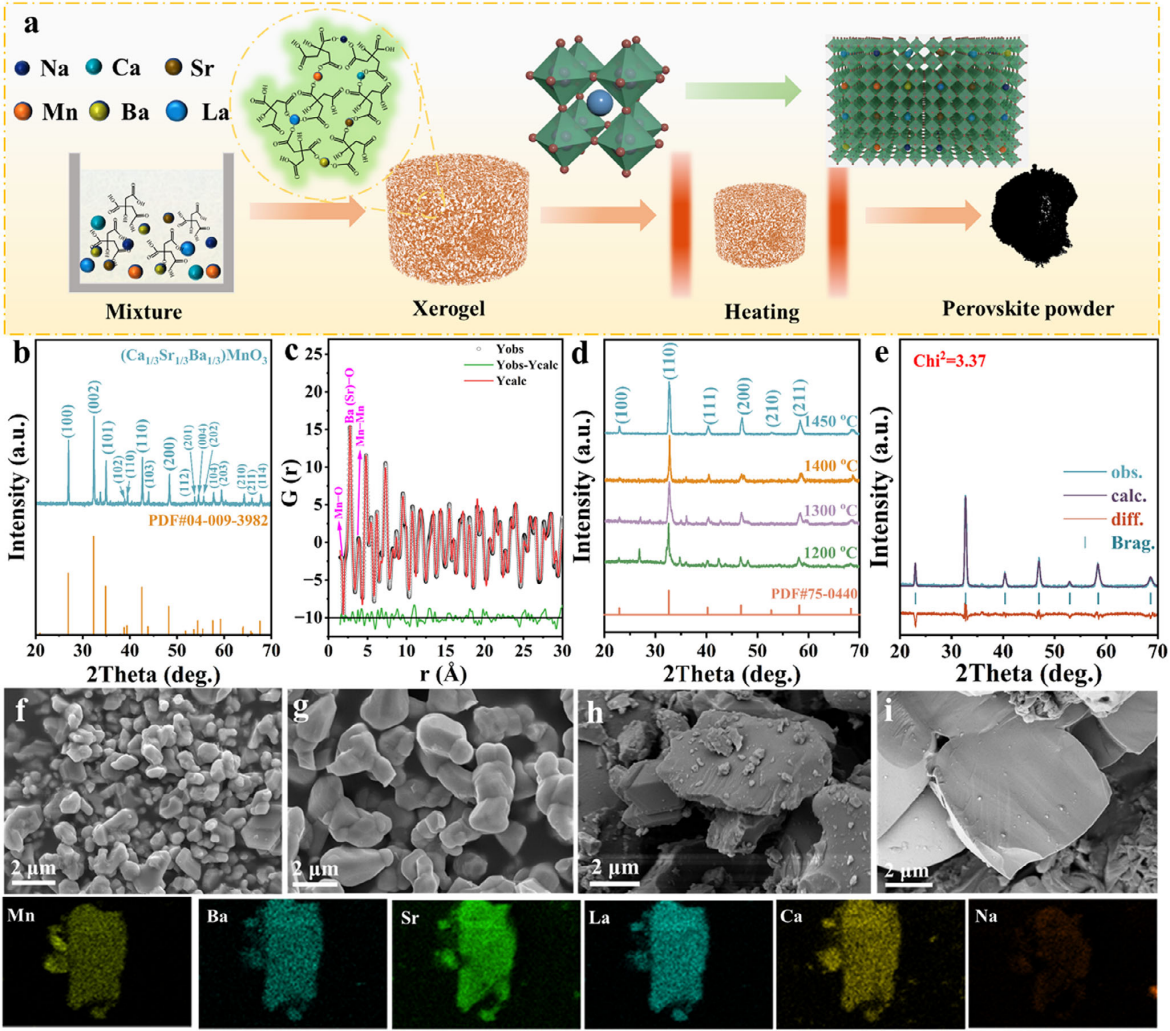
a) Schematic of the experimental process; b) XRD patterns and c) PDF fit of synchrotron‐radiation X‐ray total scattering obtained at 300 K for medium‐entropy (Ba_1/3_Sr_1/3_Ca_1/3_)MnO_3_; d) XRD patterns and e) XRD refinement pattern of high‐entropy (La_0.2_Ba_0.2_Sr_0.2_Ca_0.2_Na_0.2_)MnO_3_ samples calcinated at different temperatures; SEM images of f) SrMnO_3_, g) (Ba_1/3_Sr_1/3_Ca_1/3_)MnO_3_, h) (La_0.25_Ba_0.25_Sr_0.25_Ca_0.25_)MnO_3_, and (i) (La_0.2_Ba_0.2_Sr_0.2_Ca_0.2_Na_0.2_)MnO_3_ with elemental mapping.

The X‐ray diffraction (XRD) patterns of the as‐synthesized medium‐ and high‐entropy samples, which exhibit significant distinctions, are shown in **Figure** **1**b–d and S1 (Supporting Information). The radius and valence state of the A‐site cation influence the perovskite structure. Thus, alkali earth metals (Ca, Sr, and Ba) with different ionic radii were introduced into the A‐sites, followed by elements (La and Na) with similar radii but varying valence states. The medium‐entropy (Ba_1/3_Sr_1/3_Ca_1/3_)MnO_3_ tends to form a hexagonal structure belonging to space groups *P*63/*mmc* (164) with cell parameters *a* = *b* = 5.51 Å, *c* = 9.10 Å and *Pnma* with cell parameters *a* = 5.31 Å, *b* = 7.49 Å, *c* = 5.52 Å, as shown in the Rietveld neutron diffraction pattern in Figure S1a (Supporting Information). The pair distribution function (PDF) fit of the synchrotron‐radiation X‐ray total scattering obtained at 300 K for the medium‐entropy (Ba_1/3_Sr_1/3_Ca_1/3_)MnO_3_ is shown in Figure 1b. The PDF results show that the proportion of *P*63/*mmc* is ≈87.2%. The two‐phase ratio results are significantly different from those of the neutron diffraction pattern. Thus, a local distortion inside the material can be predicted, causing it to have a hexagonal structure in the long‐range order. Local distortions may lead to differences in the crystal structures of the materials. In contrast, the medium‐entropy (La_0.25_Ba_0.25_Sr_0.25_Ca_0.25_)MnO_3_ sample displays impurity phases, as shown in the diffractogram in Figure S1b (Supporting Information), which persist even with an increasing sintering temperature. Interestingly, when the five elements are equimolarly immolated into the A‐site to form (La_0.2_Ba_0.2_Sr_0.2_Ca_0.2_Na_0.2_)MnO_3_, the diffraction peaks (Figure 1d) exhibit symmetry and sharpness with increasing temperature, indicating the formation of single‐phase well‐crystallized crystals. Rietveld analysis (Figure 1e) suggests a cubic perovskite (*a* = *b* = *c* = 3.84 Å) with space group *Pm* $\bar{3}$ *m*, with diffraction peaks at 20°–70° corresponding to the cubic perovskite planes (100), (110), (111), (200), (210), (211), (220). Figure S2 (Supporting Information) presents the diffractograms of AMnO_3_ (*A* = La, Ba, Sr, Ca, Na) showing significant differences in crystal structure; however, (La_0.2_Ba_0.2_Sr_0.2_Ca_0.2_Na_0.2_)MnO_3_ crystallizes completely as a single cubic structure, despite solid solutions with different crystal structures.

The main differences in the crystal structures among the three samples stem from the radii of the cations at the A‐ and B‐sites in the perovskite structures, as proposed by *Goldschmidt's* tolerance factor (*t*):^[^ ^31^ ^]^

(1) $$t=\frac{R_{A}+R_{O}}{\sqrt{2}(R_{B}+R_{O})}\;$$

where *R_A_*, *R_B_*, and *R_O_* represent the radii of the cations at the A‐sites, B‐sites, and oxide ions, respectively. Generally, a stable perovskite structure corresponds to a *t* value ranging from 0.77 to 1.10. Perovskites tend to form cubic structures for *t*≈1, orthorhombic or rhombohedral structures for *t* <0.9, and tetragonal or hexagonal structures for *t* >1.^[^ ^32^ ^]^ The cation radii and *t* values of the ceramics are listed in Table S1 (Supporting Information). The calculated *t* values do not completely align with the experimental results. Generally, *t* values decrease with the introduction of La and Na, favoring the tetragonal phase. However, the experimental results of high‐entropy ceramics show cubic or quasi‐cubic structures, indicating that high‐entropy ceramics, with their complex internal chemical environments and differences among multiple elements (chemical properties and ionic radii), can expand the solid–solution boundary of the materials. The introduction of La and Na as heterovalent ions into the perovskite also satisfies the charge balance, as represented by A^2+^B^4+^O_3_.

The nonstoichiometry of oxygen in the crystal structure significantly affects the octahedral arrangement, resulting in a lower structural symmetry.^[^ ^33^ ^]^ The charge‐compensation mechanism of Mn ions (Mn^n+^ where *n* = 2, 3, and 4) is activated when La and Na of different valence states are introduced. The intrinsic charge‐compensation mechanism can stabilize the situation in which the A‐site cations are partially substituted by multivalent cations (La^3+^ and Na^1+^); however, compensation is not stimulated if the cation is replaced with only La^3+^.^[^ ^34^ ^]^ Furthermore, sufficient entropy‐driven mechanisms reduce the tendency for ordering and segregation in high‐entropy perovskites.^[^ ^35^ ^]^ The effective coordination numbers (based on Hoppe's formula) of the elements at the A and B‐sites display the same tendency as the *t* values.^[^ ^36^ ^]^ The B‐site cations approximate ideal octahedral coordination regardless of the type of cations in the perovskite. However, the cationic coordination at the A‐sites deviates significantly from the ideal 12‐fold coordination with smaller cations added or replaced at the A‐site, causing the effective coordination number to deviate from the ideal 12‐coordination.

Figure 1f–i shows SEM images of the specimens with increasing entropy, and Figure S3 (Supporting Information) shows graphics and energy‐dispersive spectroscopy (EDS) maps, indicating a homogeneous distribution of elements without segregation in SrMnO_3_ and (La_0.2_Ba_0.2_Sr_0.2_Ca_0.2_Na_0.2_)MnO_3_. However, element segregation is observed in (Ba_1/3_Sr_1/3_Ca_1/3_)MnO_3_ and (La_0.25_Ba_0.25_Sr_0.25_Ca_0.25_)MnO_3_ (Figure S3, Supporting Information). Figure S4 (Supporting Information) reveals that the high‐entropy perovskite particles grow larger with increasing sintering temperature. Heterogeneous particles tend to form cuboid or polyhedral morphologies with obvious grain boundaries. At higher temperatures, the edges and corners gradually blur or disappear, forming circular particles. The higher the sintering temperature, the stronger the atomic‐diffusion energy, which accelerates the formation and growth of sintering necks between particles.^[^ ^37^, ^38^ ^]^ This reduces the variation in the growth velocity along different directions, ultimately resulting in the spheroidization of the particles.

**Figure 2:**
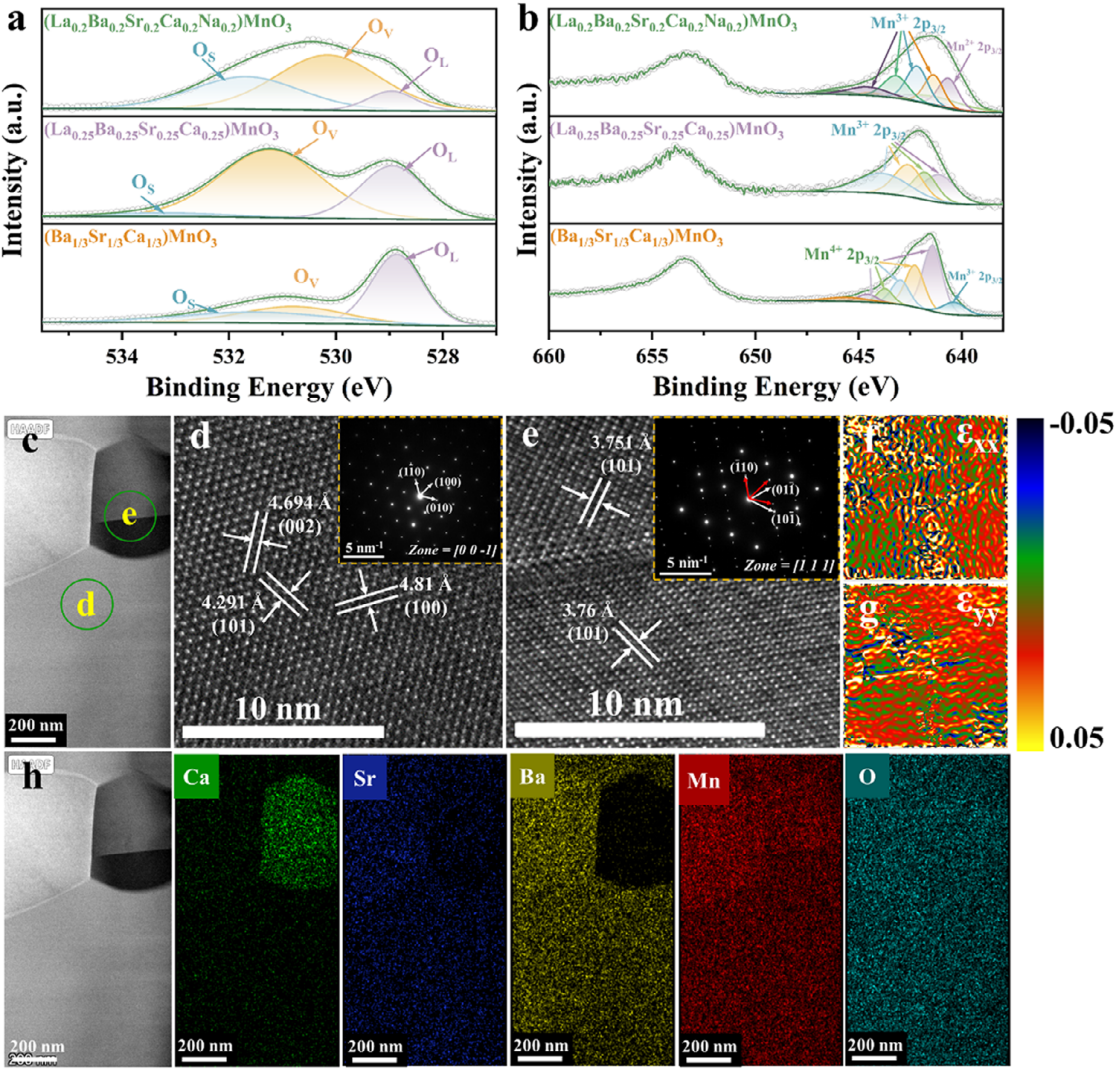
XPS spectra of a) O and b) Mn ions with increasing entropy, c) HAADF of (Ba_1/3_Sr_1/3_Ca_1/3_)MnO_3_, d,e) SAED and high‐resolution images for different regions in (c), f,g) the corresponding strain field ε_xx_ and ε_yy_ of the TEM image was obtained by the GPA method, andh) corresponding elemental distribution of Ca, Sr, Ba, Mn, and O in (c).

The changes in the valence states of the oxygen vacancies and Mn ions are elucidated through X‐ray photoelectron spectroscopy (XPS) spectra, providing an intuitive insight into these variations. The results confirmed that multiple cations are present in the perovskites (**Figure** **2**a,b; Figure S5, Supporting Information). Figure 2a,b illustrates the fine spectra of the Mn and O ions in the three specimens, respectively. Large quantities of Mn^4+^ and Mn^3+^ valences exist in (Ba_1/3_Sr_1/3_Ca_1/3_)MnO_3_ and (La_0.25_Ba_0.25_Sr_0.25_Ca_0.25_)MnO_3_, whereas both Mn^2+^ and Mn^3+^ valences exist in (La_0.2_Ba_0.2_Sr_0.2_Ca_0.2_Na_0.2_)MnO_3_, implying the spin‐orbit splitting of Mn ions. As shown in Figure 2b, the Mn ion 2p_3/2_ orbit contains a variety of splitting peaks: Mn^2+^ possess six peaks at 640.3, 641.5, 642.3, 643.2, 647.5, and 645.0 eV; Mn^3+^ has five peaks at 641.7, 642.4, 643.2, 644.2, and 645.8 eV; and Mn^4+^ has five peaks at 642.5, 643.5, 644.3, 645.3, and 646.4 eV.^[^ ^39^ ^]^ Combined with the XPS spectrum of O, shown in Figure 2a, three main spectral peaks are observed: lattice oxygen (O_L_) at ≈529 eV, vacancy oxygen (O_V_) at 531 eV, and adsorbed oxygen (O_S_) at 532 eV.^[^ ^33^ ^]^

In (Ba_1/3_Sr_1/3_Ca_1/3_)MnO_3_, the A‐site cations (Ca, Sr, Ba) are all in the + 2 valence state, while Mn ions are mainly in the + 4 valence state to achieve charge balance. However, the XPS spectra in Figure 2a indicate the presence of a small amount of Mn^3+^ due to oxygen vacancies. Owing to the introduction of La^3+^ and the corresponding increase in oxygen vacancies, as shown in Figure 2, Mn^3+^ cannot maintain the charge balance. In high‐entropy perovskites, Na^+^ can effectively balance the charge and activate the charge‐compensation mechanism of manganese ions. XPS analysis indicates that the ratio of Mn^2+^ to Mn^3+^ in (La_0.2_Ba_0.2_Sr_0.2_Ca_0.2_Na_0.2_)MnO_3_ is ≈42.8:57.2 (shown in Figure 2b), suggesting the significant formation of oxygen vacancies. The spin‐orbit splitting of manganese and the generation of oxygen vacancies influence the crystal structure and properties of high‐entropy perovskites.^[^ ^40^ ^]^

Transmission electron microscopy (TEM) was performed to further investigate the morphology of (Ba_1/3_Sr_1/3_Ca_1/3_)MnO_3_, as shown in Figure 2c–e. Figure 2c illustrates the nanoscale morphology of (Ba_1/3_Sr_1/3_Ca_1/3_)MnO_3_ and the selected‐area electron diffraction (SAED) patterns of two different areas exhibited in Figure 2d,e. The diffraction spots corresponding to the crystal faces along the [001]‐zone axes in Figure 2d match the XRD pattern in Figure 1b (PDF#04‐009‐3982), whereas those along the [111] axis in Figure 2e align well with the unidentified diffraction peaks in Figure 1b (PDF#00‐050‐1748). The high‐resolution imaging reveals distinct lattice parameters, with the former having a maximum interplanar spacing of 4.694 Å for the (002) plane, and the latter having a spacing of 3.76 Å for the (101) plane, which is consistent with the XRD results. Figure 2f,g shows the high‐resolution TEM images of Figure 2f obtained using the geometric phase analysis (GPA) method. The image shows a relatively obvious strain, which confirms that significant strain exists in the materials. The element mapping (Figure 2h) confirms serious segregation of the Ca element; although Ca and Ba occupy the same lattice sites, they cannot form a solid solution owing to the substantial difference in their ionic radii. This leads to Ca segregation, particularly at lower temperatures, which is consistent with the XRD results.

**Figure 3:**
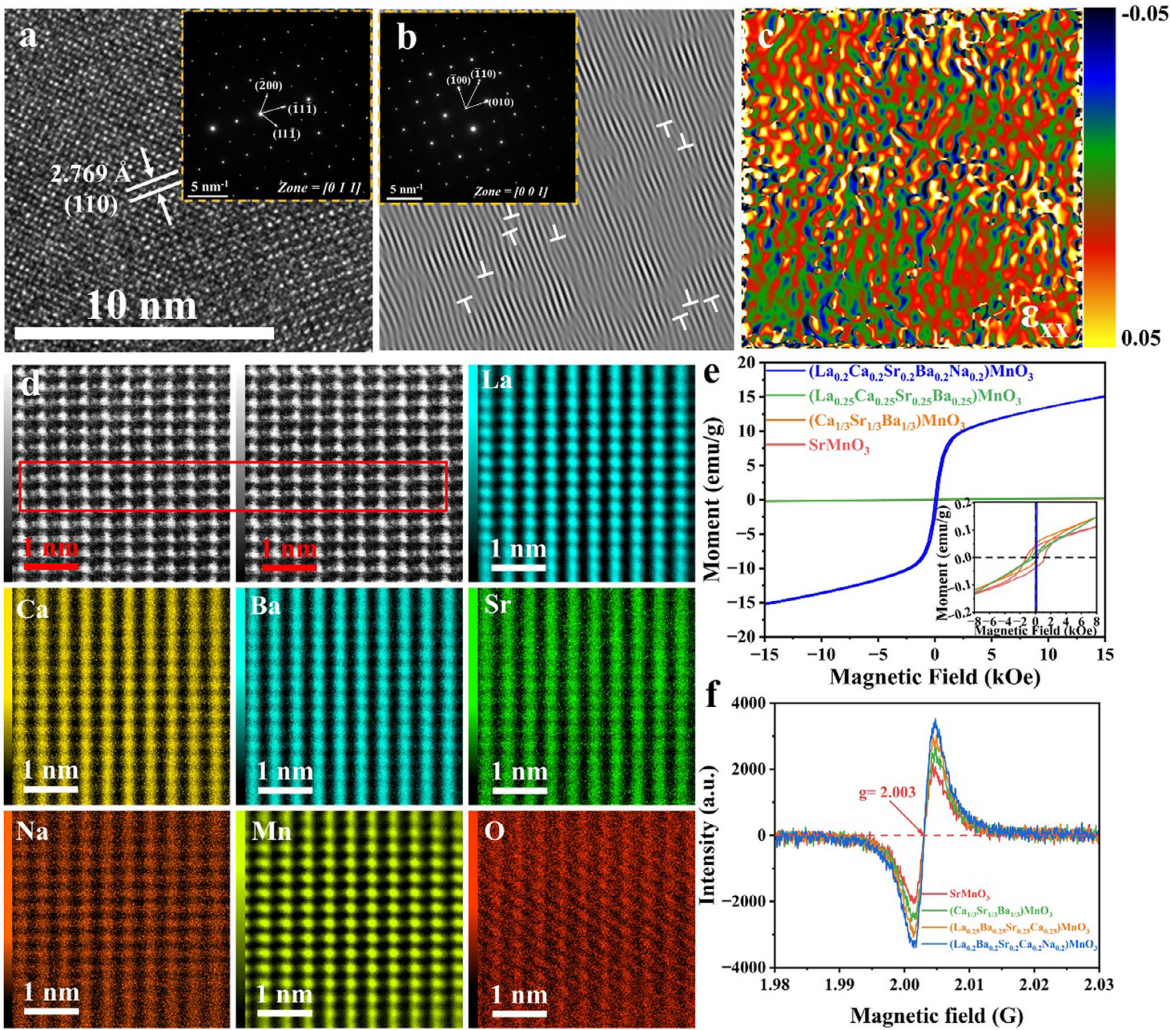
a) SEAD on the [011] axis and high‐resolution images; b) SEAD on [001] zone axes (top left corner) and the iFFT images; c) the corresponding strain field ε_xx_ of TEM image was obtained by GPA method,d) HAADF‐STEM and corresponding elemental distribution of high‐entropy ceramics (La_0.2_Ba_0.2_Sr_0.2_Ca_0.2_Na_0.2_)MnO_3_; e) magnetization‐hysteresis loops (M‐H) curve of the specimens at room temperature, and f) the EPR curve of a high‐entropy ceramic.

High‐resolution TEM and SAED analyses of (La_0.2_Ba_0.2_Sr_0.2_Ca_0.2_Na_0.2_)MnO_3_ along the [011] and [100] axes are shown in **Figure** **3**a–d. In Figure 3a, the crystal plane spacings of 3.966 and 2.769 Å correspond to the (100) and (110) planes, respectively, which align with XRD data in Figure 1h. The relevant inverse fast Fourier transform (iFFT) image is illustrated in Figure 3b, where abundant dislocations are observed, indicating lattice distortion and strain stemming from cations of different sizes and masses existing in the high‐entropy ceramics. Such distortions and defects are typical in high‐entropy ceramics and influence the internal electron migration and response to external fields.^[^ ^18^ ^]^ The SEAD patterns in Figure 3a,b further confirm the successful synthesis of high‐entropy perovskites. Furthermore, the GPA analysis of the high‐resolution images in Figure 3c shows severe strain within the high‐entropy material. This further indicates the presence of abundant defects inside high‐entropy perovskites, which will likely further promote the dissipation of electromagnetic waves by high‐entropy materials. In contrast to high‐entropy materials, the lattice structure in medium‐entropy materials, such as (Ba_1/3_Sr_1/3_Ca_1/3_)MnO_3_, is relatively regular and maintains a crystal structure similar to that of AMnO_3_. This observation also considers the impact of the metal‐ion radius on the structure factor *t*. The types and distribution of elements in these materials are relatively limited, resulting in low‐entropy effects for mesentropic materials. Consequently, changes in lattice strain are primarily influenced by external stresses and the inherent properties of the material, such as lattice constants and the strength of chemical bonds. In (La_0.2_Ba_0.2_Sr_0.2_Ca_0.2_Na_0.2_)MnO_3_, variations in atomic radius can lead to different degrees of lattice distortion (lattice strain). Moreover, an increase in the entropy enhances the tolerance for lattice distortion and facilitates larger crystal‐structure transformations, thereby increasing the internal strain. Additionally, the corresponding elemental distributions of TEM (Figure S6a, Supporting Information) and HAADF‐STEM (Figure 3d) reveal no elemental segregation, further indicating the successful synthesis of homogeneous cubic high‐entropy perovskites. The linear sweep results for the elements indicated by the red box are shown in Figure S6b (Supporting Information). These results indicate that the five cations successfully occupy the same lattice site. The GPA analysis (Figure S6c, Supporting Information) of the high‐entropy ceramics indicates varying degrees of stress and strain between atoms, which intensifies the complexity within the local structure, hinders the movement of carriers in the crystal, and affects the polarization effect of the material.

### 2.2 Magnetism

The A‐site cations in the perovskite structures influence the valence states of the Mn ions, as confirmed by the XPS spectra. To investigate the magnetic properties and their dependence on the Mn ions, magnetization‐hysteresis loops (M‐H) were measured at room temperature (Figure 3e), along with the temperature‐dependent magnetization (M‐T) curves of the high‐entropy ceramics (Figure S7, Supporting Information). The M‐H curves of high‐entropy perovskites (La_0.2_Ba_0.2_Sr_0.2_Ca_0.2_Na_0.2_)MnO_3_ exhibit typical “S” shapes, indicating their ferromagnetic behavior. The saturation magnetization and coercivity of the other samples are very low, indicating that they may exhibit quasi‐superparamagnetic behavior. The electron paramagnetic resonance (EPR) curves of each sample with respect to the oxygen vacancies are shown in Figure 3f. Moreover, *g* = 2.003 confirms the existence of oxygen vacancies in the material. As the entropy in the samples continuously increases, the signal intensity of the oxygen vacancies gradually strengthens.

According to crystal field theory, Mn ions, as the center of the octahedral structure in perovskites, experience a crystal field that splits the d‐orbitals into a double degenerate e_g_ orbital with higher energy and a triple degenerate t_2g_ orbital with lower energy. The XPS results for SrMnO_3_ indicate that Mn mainly exhibits the + 4 valence state with an electron arrangement of t3 2ge0 g (d^3^). Under the action of a crystal field and Hund's rule, Mn^4+^−O^2−^−Mn^4+^ superexchange interactions occur, where the spin magnetic moments are antiparallel, resulting in antiferromagnetism.^[^ ^41^ ^]^ However, the presence of oxygen vacancies introduces a small amount of Mn^3+^, leading to Mn^3+^−O^2−^‐Mn^4+^ double‐exchange interactions that tend to produce ferromagnetic behavior.^[^ ^42^, ^43^ ^]^ Similar to SrMnO_3_, (Ba_1/3_Sr_1/3_Ca_1/3_)MnO_3_ exhibits weak magnetic properties with Mn^4+^ as the dominant state. (La_0.25_Ba_0.25_Sr_0.25_Ca_0.25_)MnO_3_ also shows weak magnetism, but the addition of La^3+^ increases the number of oxygen vacancies. XPS results indicate that Mn^3+^ is the predominant valence state in (La_0.2_Ba_0.2_Sr_0.2_Ca_0.2_Na_0.2_)MnO_3_. In such a case, the magnetic interaction is mainly governed by Mn^3+^−O−Mn^3+^ super‐exchange, which generally produces only weak or paramagnetic behavior.

In high‐entropy perovskites, Mn^2+^−O^2−^ Mn^3+^ interactions lead to double‐exchange effects facilitated by the O 2*p* orbitals. The electron transitions from the Mn^2+^ (t3 2ge2 g) orbital to the O 2*p* orbital and then from the O 2*p* orbital to Mn^3+^ change the electron configuration of Mn from t3 2ge1 g(d^4^) to t3 2ge2 g(d^5^) without spin reversal during the electron transition. This promotes ferromagnetism through parallel spin alignment between the Mn ions. The high‐spin e_g_ orbitals in Mn^2+^ (t3 2ge1 g d^4^) and Mn^3+^ (t3 2ge2 g d^5^) interact with ligands, leading to Jahn–Teller distortions that reduce the orbital degeneracy and symmetry, thereby lowering the system energy and stabilizing the perovskite structure.^[^ ^30^ ^]^ Additionally, the high‐entropy M‐H loop's “thin and tall” profile suggests that it is a “soft magnetic” material, capable of quickly and efficiently storing and releasing magnetic energy. (La_0.2_Ba_0.2_Sr_0.2_Ca_0.2_Na_0.2_)MnO_3_ exhibits significant magnetic enhancement. This enhancement is attributed to the high‐entropy nature of the material, which, through the disordered doping of multiple metal elements, can substantially influence its lattice structure and electronic properties, thereby affecting its magnetism. Figure 2b illustrates that in (La_0.2_Ba_0.2_Sr_0.2_Ca_0.2_Na_0.2_)MnO_3_, the presence of La^3+^, Ba^2+^, Sr^2+^, Ca^2+^, and Na^+^ ions can induce changes in the Mn^3+^/Mn^2+^ ratio when doped with various metal ions, which, in turn, impacts the magnetic characteristics of the material. Meanwhile, the presence of oxygen vacancies promotes and stabilizes the coexistence of Mn^2+^(t3 2ge2 g) and Mn^3+^(t3 2ge1 g) ions, enabling electron delocalization between these two oxidation states, thereby facilitating ferromagnetic spin alignment. Oxygen vacancies also affect theMn^2+^−O^2−^‐Mn^3^⁺ double exchange interaction, but their concentration is effectively regulated by the high‐entropy stabilization effect. Thus, in this high‐entropy perovskite system, the enhancing role of oxygen vacancies on magnetism is predominant. Disordered doping in high‐entropy materials can also lead to lattice distortion and influence the hyperexchange coupling between the Mn ions. Furthermore, the introduction of Na^+^ facilitates the mixing of Mn 3d electrons. Given that Na^+^ has a small ionic radius, this results in greater lattice distortion, enhances the exchange interaction between Mn^3+^ and Mn^2+^, and ultimately improves the magnetic response of the material.

The M‐T curves of the high‐entropy perovskites shown in Figure S7 (Supporting Information) were obtained under a magnetic field of 200 Oe. The field cooling (FC) and zero‐field cooling (ZFC) curves coincide between 5 and 400 K, with divergence at 50 K. The magnetic susceptibility χ was plotted in Figure S7 (Supporting Information) as a function of temperature with the Curie temperature *Tc* = 369.2 K determined by the interpolation and extrapolation method.

### 2.3 Microwave Absorption

**Figure 4:**
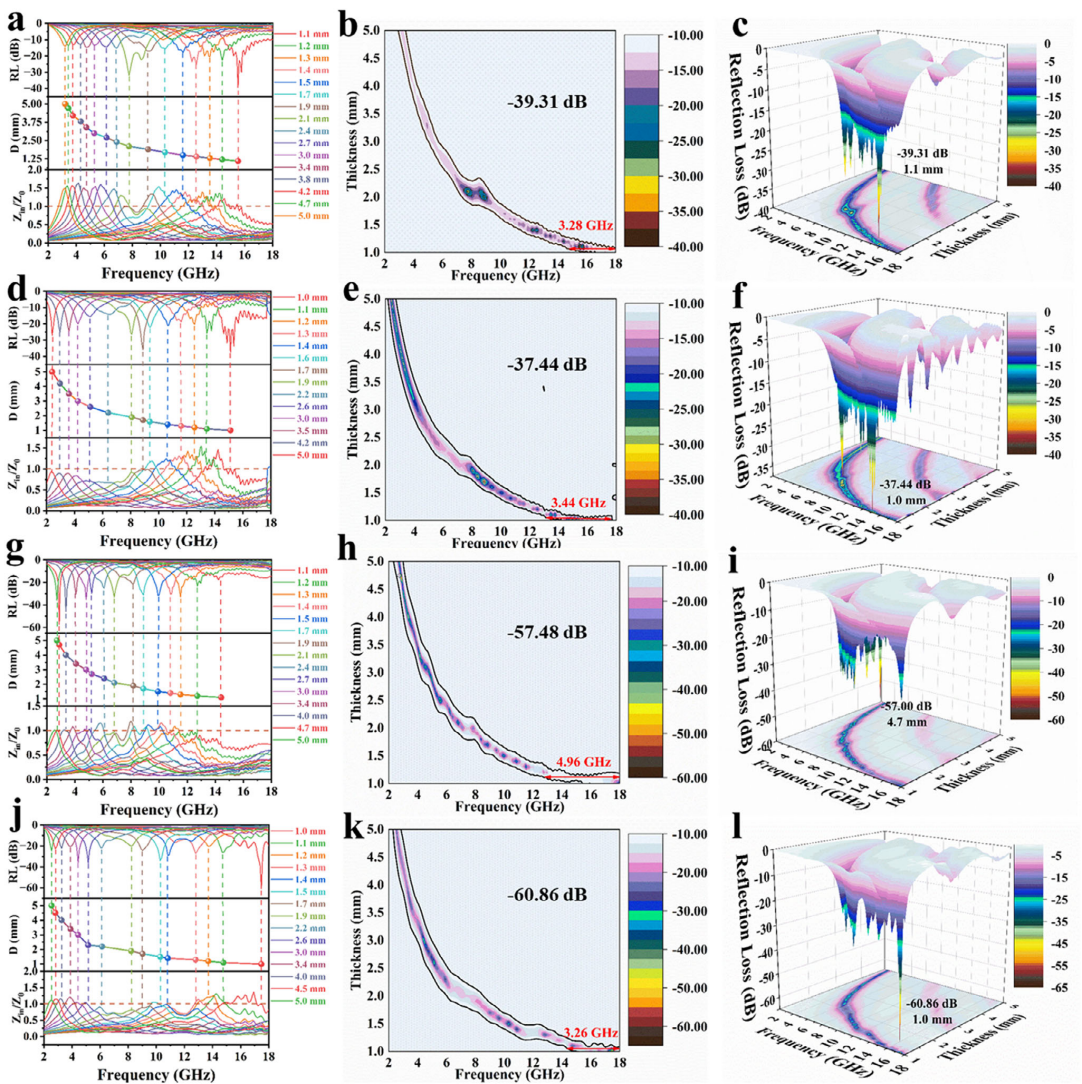
Reflection loss, thickness, and impedance matching with frequencies of high‐entropy ceramics at various temperatures and corresponding 2D and 3D diagrams of reflection loss at different temperatures: a–c) 1200 °C, d–f) 1300 °C, g–i)1400 °C, and j–l) 1450 °C.

The relevant parameters of the specimen at 2‒18 GHz were obtained using a network vector analyzer, and the absorption performance of the obtained samples was evaluated based on the reflection loss (RL):^[^ ^44^, ^45^ ^]^

(2) $$\mathrm{RL}=20\mathrm{lg}\left|\frac{Z_{\mathrm{in}}-Z_{0}}{Z_{\mathrm{in}}+Z_{0}}\right|$$

(3) $$Z_{in}=Z_{0}\sqrt{\frac{\mu_{r}}{\varepsilon_{r}}\tanh\left[j\left(\frac{2\pi fd}{c}\right)\sqrt{\mu_{r}\varepsilon_{r}}\right]}$$

(4) $$\alpha =\frac{\sqrt{2}\pi f}{c}\times \sqrt{\left(\mu^{\prime \prime }\varepsilon^{\prime \prime }-\mu^{\prime }\varepsilon^{\prime }\right)+\sqrt{{\left(\mu^{\prime \prime }\varepsilon^{\prime \prime }-\mu^{\prime }\varepsilon^{\prime }\right)}^{2}+{\left(\mu^{\prime }\varepsilon^{\prime \prime }+\mu^{\prime \prime }\varepsilon^{\prime }\right)}^{2}}}$$

where *Z_0_* and *Z_in_* symbolize the impedance in free space and input characteristic impedance, respectively, *ε_r_* denotes the complex permittivity, *µ_r_* is the permeability, *f* and *d* represent the frequency and sample thickness, respectively, and *c* is a constant. **Figure** **4** and Figures S8 and S9 (Supporting Information) shows the reflection loss of high‐entropy materials, AMnO_3_, and medium‐entropy ceramics, (Ba_1/3_Sr_1/3_Ca_1/3_)MnO_3_ and (La_0.25_Ba_0.25_Sr_0.25_Ca_0.25_)MnO_3_, at room temperature, along with the corresponding impedance matching diagrams. In Figure 4, the minimum reflection loss (RL_min_) and effective absorption bandwidth (EAB) values of the sample at different temperatures are −39.31 dB (1.1 mm), −37.44 dB (1.0 mm), −57.00 dB (4.7 mm), and −60.86 dB (1.0 mm) and 3.28, 3.44, 4.96, and 3.26 GHz respectively. Figure S8 (Supporting Information) shows the RL_min_ values of LaMnO_3_, BaMnO_3_, SrMnO_3_, and CaMnO_3_ as well as impedance matching, where LaMnO_3_ and SrMnO_3_ achieve RL_min_ values below −10 dB for thicker samples. Figure S9 (Supporting Information) shows the reflection losses for (Ba_1/3_Sr_1/3_Ca_1/3_)MnO_3_ and (La_0.25_Ba_0.25_Sr_0.25_Ca_0.25_)MnO_3_ with thicknesses ranging from 1 to 10 mm. For (La_0.25_Ba_0.25_Sr_0.25_Ca_0.25_)MnO_3_, the RL_min_ value of −44.59 dB is achieved at 8.8 mm. The reflection‐loss mapping curve in Figure 4 shows that the effective absorbing region shifts to a lower frequency as the thickness increases, following the 1/4 wavelength rule.^[^ ^46^ ^]^ Additionally, Figure 4 illustrates that the impedance matching (*Z*  = *Z_in_* /*Z*_0_) of high‐entropy samples is concentrated within the range of 0.8–1.5, enabling effective absorption of electromagnetic waves from the air into the material. High‐entropy perovskites exhibit superior electromagnetic‐wave absorption capabilities compared to simple Mn‐matrix perovskites and medium‐entropy perovskites.

Electromagnetic waves comprise alternating electric and magnetic fields. When exposed to a dynamic energy field, the dielectric and magnetic properties of materials tend to lag behind the electromagnetic field.^[^ ^47^ ^]^ RL is the evaluation of the overall electromagnetic‐wave absorption properties of a material, with permittivity and permeability being the key indicators of the underlying absorption mechanisms.

**Figure 5:**
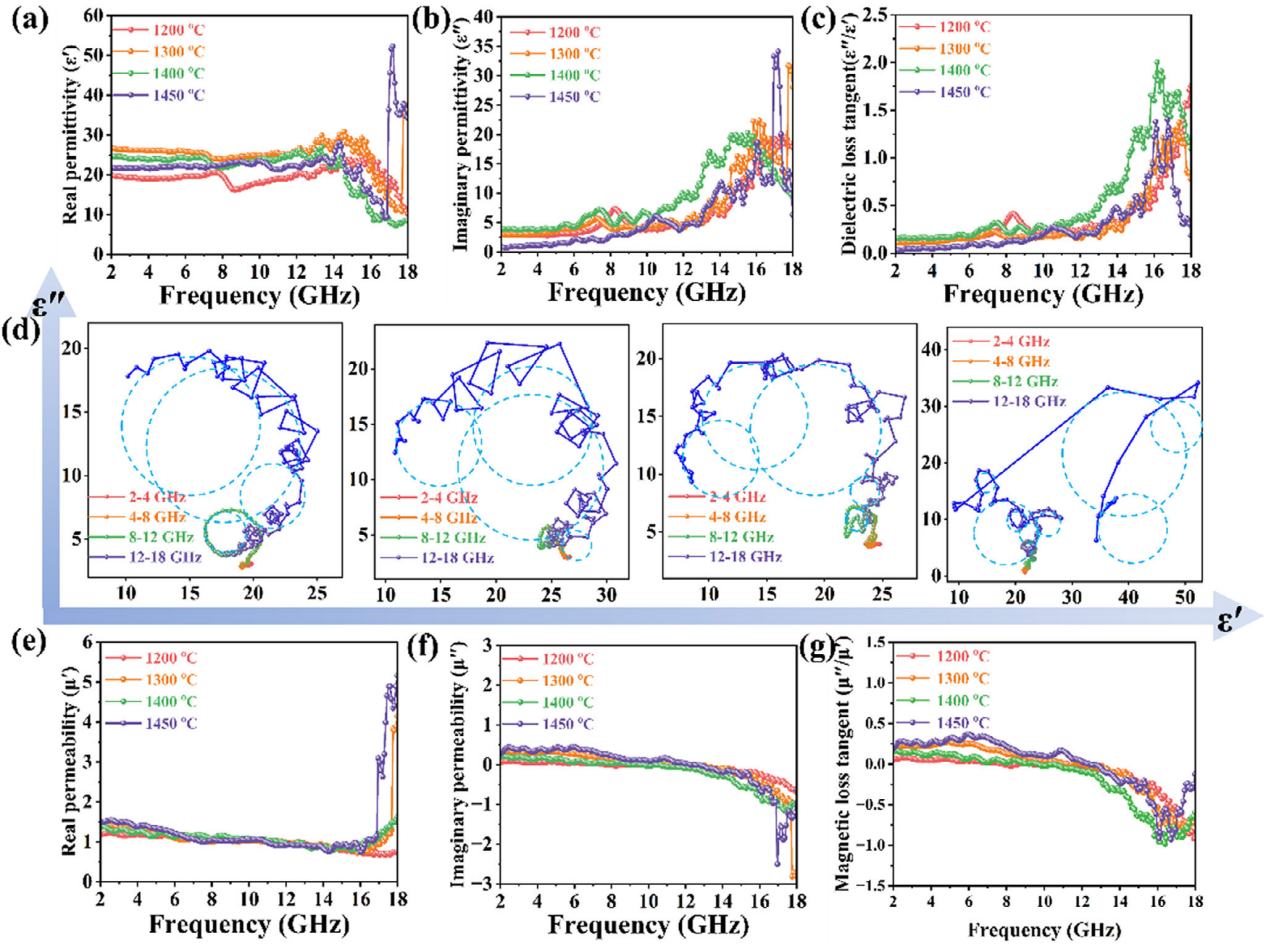
a) Real permittivity (εʹ), b) imaginary permittivity (ε″), c) dielectric‐loss tangent (ε″/εʹ), d) Cole–Cole circle, e) real permeability (μʹ),f) imaginary permeability (μ″), and g) magnetic‐loss tangent (μ″/μʹ) of high‐entropy ceramics at various temperatures.

The dielectric and magnetic properties are represented by complex parameters: the real part of the permittivity represents the storage capacity of the material, whereas the imaginary part reflects the loss capacity. These parameters for high‐entropy ceramics and corresponding Mn‐based perovskite ceramics, including the real permittivity εʹ, imaginary permittivity εʺ, and dielectric‐loss tangent value tan(εʺ/εʹ), are shown in **Figure** **5**a–c and S10a–c (Supporting Information). At low frequencies, εʹ indicates subtle changes, indicating the storage ability of high‐entropy ceramics. However, at higher frequencies, the induced charge of the material cannot keep pace with the rapidly changing electric field, resulting in instantaneous polarization. The swift variation in charge under high‐frequency electromagnetic fields leads to electron vibrations and transitions, thereby reducing the charge‐storage capacity of the material.

Compared with high‐entropy perovskites, medium‐entropy and the corresponding Mn‐based terpolymer perovskites have lower dielectric parameters, as well as reduced storage and loss capacities for electromagnetic waves. Therefore, larger sample thicknesses are required to extend the path of the electromagnetic waves within the material to achieve efficient absorption (RL<−10 dB). The dielectric properties of these samples can be regulated using entropy engineering and the high‐entropy effect, which alters the internal structures of the materials. Both permittivity and the loss tangent are frequency‐dependent, with electron polarization, dipole polarization, atomic polarization, and ion polarization contributing to the dielectric loss at various frequencies.^[^ ^48^ ^]^

At low frequencies, dipoles can respond to electromagnetic waves in real time, leading to low loss capacity and a small εʺ. As frequency increases, the ability of the material to dissipate the electromagnetic wave improves, causing ε" to increase significantly. This is attributed to the electron‐hopping frequency between the metal cations, which matches the frequency of the incident electromagnetic wave. As shown in Figure S4 (Supporting Information), the particle size gradually increases with increasing firing temperature, but the dielectric loss parameters of the high‐entropy ceramics at different firing temperatures show similar changing trends and dielectric‐loss values. Small high‐entropy ceramic particles can enhance dielectric loss to some extent. Furthermore, Figure 1f–i indicate that the particle size of (La_0.2_Ba_0.2_Sr_0.2_Ca_0.2_Na_0.2_)MnO_3_ is ≈7 µm, while that of SrMnO_3_ is only in the nanometer scale. Despite its smaller particle size, SrMnO_3_ exhibits a low dielectric loss. This finding suggests that the influence of the particle size on the dielectric loss of materials is limited. In the electromagnetic‐frequency band, dipole relaxation in response to varying electromagnetic frequencies is an important factor in dielectric loss, which is often explained by the Debye relaxation:^[^ ^49^ ^]^

(5) $$\varepsilon^{\prime }=\varepsilon_{\infty }+\frac{\varepsilon_{s}-\varepsilon_{\infty }}{{\left(2\pi f\right)}^{2}\tau^{2}+1}$$

(6) $$\varepsilon^{\prime \prime }=\frac{\varepsilon_{s}-\varepsilon_{\infty }}{{\left(2\pi f\right)}^{2}\tau^{2}+1}+\frac{\sigma }{\varepsilon_{0}}$$

(7) $${\left(\varepsilon^{\prime }-\frac{\varepsilon_{s}-\varepsilon_{\infty }}{2}\right)}^{2}+{\left(\varepsilon^{\prime \prime }\right)}^{2}={\left(\frac{\varepsilon_{s}-\varepsilon_{\infty }}{2}\right)}^{2}\;$$

where ε_*s*_ represents static permittivity, τ denotes the relaxation time, and ε_∞_ is the permittivity at the high‐frequency limit. Cole–Cole circles were drawn using the relationship between the real and imaginary parts of the permittivity provided by the above formula, as shown in Figure 5d and Figure S11 (Supporting Information). Continuous curvature changes indicate the existence of multiple relaxation mechanisms. For the high‐entropy perovskite materials, the plots do not exhibit a perfect semicircular shape; instead, they display a concave and asymmetric arc. This behavior indicates a continuous distribution of relaxation times within the material. The presence of numerous defects in the high‐entropy perovskite, along with local internal stress caused by lattice distortion, leads to electron accumulation at defects and grain boundaries. Additionally, the random distribution of A‐site cations with varying radii and oxidation states creates a diverse local environment. Each environment induces polarization loss at slightly different characteristic frequencies, resulting in the observed broad and overlapping relaxation behavior.

For the high‐entropy perovskite materials, the plots do not exhibit a perfect semicircular shape; instead, they display a concave and asymmetric arc. This behavior indicates a continuous distribution of relaxation times within the material. The presence of numerous defects in the high‐entropy perovskite, along with local internal stress caused by lattice distortion, leads to electron accumulation at defects and grain boundaries.^[^ ^50^, ^51^ ^]^ Additionally, the random distribution of A‐site cations with varying radii and oxidation states creates a diverse local environment. Each environment induces polarization loss at slightly different characteristic frequencies, resulting in the observed broad and overlapping relaxation behavior.^[^ ^52^, ^53^ ^]^ The radius and oxidation state of the A‐site cation vary, which creates a series of polar dipoles. Electron hopping between manganese ions with different oxidation states further drives the dynamic behavior of these dipoles. Within the ceramic samples, numerous grain boundaries are formed, where space charge accumulates at the interfaces, leading to interfacial polarization and relaxation. Simultaneously, at lower frequencies, perovskite materials exhibit some degree of conductivity loss owing to the movement of charge carriers. The coexistence of multiple dipoles in high‐entropy materials leads to polarization relaxations across various frequency bands, collectively resulting in broadband absorption. This phenomenon is the primary reason why high‐entropy designs outperform simple perovskites.

The XRD results indicate that the perovskite structure gradually transitions from a hexagonal phase to a cubic phase as different metal cations occupy the A‐site. The ability of perovskites to attenuate electromagnetic waves varies significantly depending on their crystal structures. Cubic structures may exhibit distinct electromagnetic‐wave responses depending on the propagation direction. Additionally, defects in the crystal structure of high‐entropy materials, such as distortions and vacancies, lead to localized changes in electron density. These changes can result in local electron resonance or enhanced electric‐field effects, which contribute to improved material absorption. The results indicate that hexagonal materials with a long *c*‐axis are within the high‐frequency microwave‐ and millimeter‐wave ranges. Additionally, when combined with SEM, the distribution direction and arrangement of the hexagonal layers appear to be random. A significant gap between the particles is observed, which may create a large channel within the ring that facilitates the passage of electromagnetic waves. Consequently, greater thickness is required to extend the path of electromagnetic waves within the material, thereby enhancing its ability to absorb these waves effectively.

Figure 5e–g and Figure S10d–f (Supporting Information) displays the resonance magnetic spectra of the permeability over a frequency range of 2–18 GHz, corresponding to natural resonance phenomena. The real permeability reflects the energy‐storage density in an alternating magnetic field, and is typically <1 within this frequency range.^[^ ^54^ ^]^ Owing to the imaginary part of the complex permeability μ″, the magnetic induction lags behind the applied magnetic field, leading to continuous energy consumption, specifically, the dissipation of electromagnetic waves during alternating magnetization. The hysteresis‐related energy loss is a consequence of local stress from lattice distortion, generated by elements of different radii in high‐entropy structures and domain wall “pinning” owing to internal point defects.^[^ ^55^ ^]^ The complex permeability of the high‐entropy perovskites is slightly higher than that of the Mn‐based terpolymer perovskites, although both exhibit a similar decreasing trend with increasing frequency. At higher frequencies (>12 GHz), the imaginary part of the permeability has a negative value, indicating a competitive interaction between the dielectric and magnetic losses within the material, which correlates with the increase in complex permittivity at higher frequencies.

Magnetic loss, which includes both eddy‐current loss and natural resonance, plays a crucial role in electromagnetic‐wave absorption. The real permeability (*μʹ*) shows frequency dispersion, decreasing as frequency increases. The loss of the material is determined by its intrinsic magnetic properties, with higher saturation magnetization and lower coercivity favoring higher permeability and stronger magnetic loss. An increased entropy tends to enhance the magnetic properties of perovskites. Therefore, materials with higher entropy values exhibit superior performance compared with the other samples (Figure 3e). However, magnetic loss is also influenced by magnetic coupling under an electromagnetic field. The eddy‐current loss coefficient can be used to assess the loss mechanism:^[^ ^46^, ^56^ ^]^

(8) $$C_{0}=\mu^{\prime \prime }\;{\left(\mu^{\prime }\right)}^{-2}f^{-1}$$

When the value of *C_0_* does not change with frequency *f*, the main mechanism of magnetic loss can be judged as eddy‐current loss. However, based on Figure S12a,b (Supporting Information), the magnetic loss in high‐entropy ceramics primarily arises from resonance loss. When the sizes of the sample or its internal particles are close to, or equal to, an integer multiple of half the wavelength of the electromagnetic wave, standing waves form within the material, leading to dimensional resonance effects that strongly absorb electromagnetic energy.^[^ ^57^ ^]^The frequency‐dependent fluctuations in electromagnetic parameters may be attributed to variations in grain size. Under electromagnetic‐wave excitation, dielectric and magnetic losses represent competing mechanisms for energy dissipation. As a result, at higher frequencies, *εʺ* increases while *μʺ* decreases or becomes negative.

**Figure 6:**
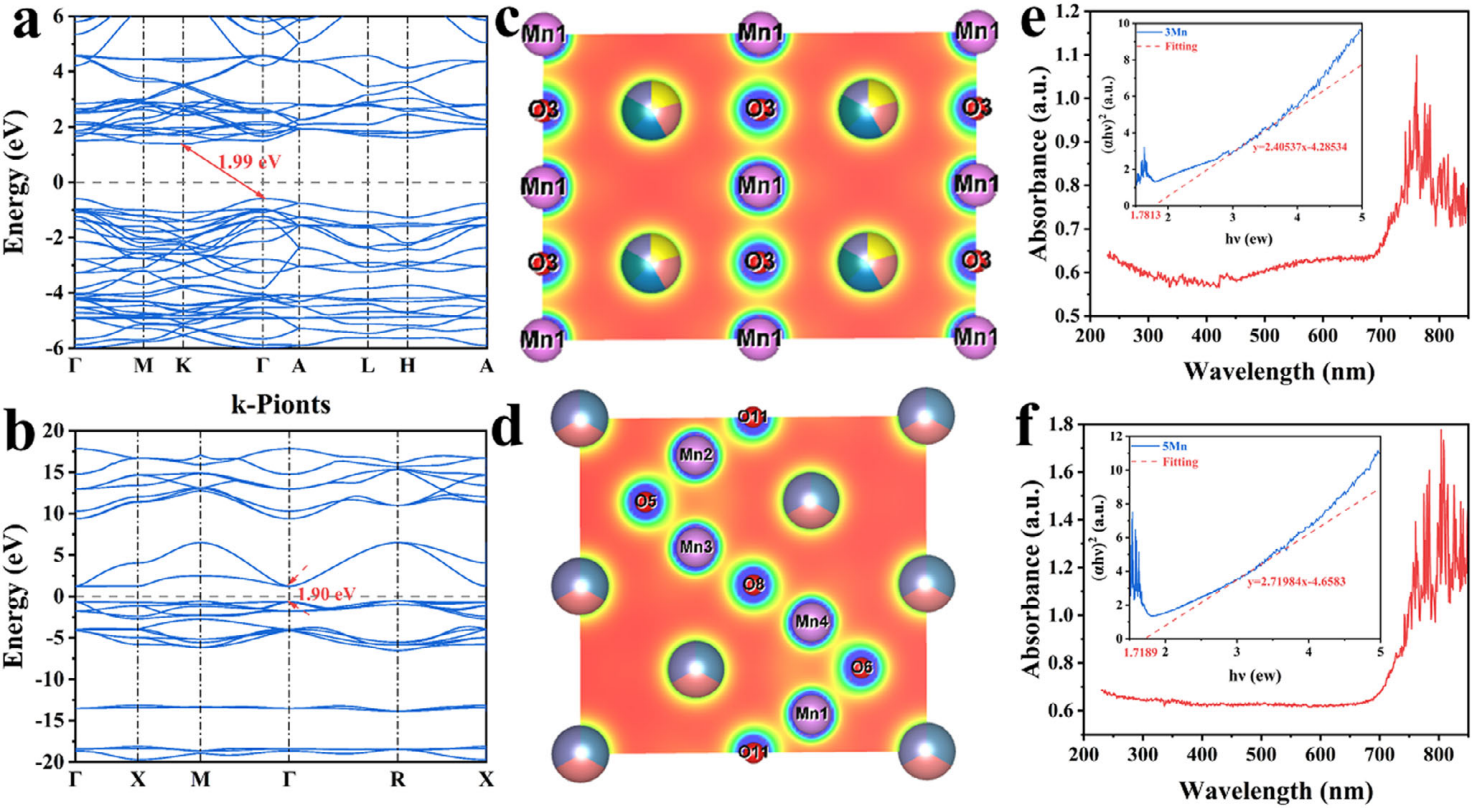
a,b) First‐principles calculations, c,d) charge‐density distribution, e,f) UV–vis absorption spectra, illustrated by *Tauc* diagram analysis, which is used to determine the bandgap width of medium‐ and high‐entropy ceramics.

The bandgaps of the medium‐ and high‐entropy perovskites were calculated using first‐principles calculations, as shown in **Figure** **6**a,b. The energy gaps *E_g_* were 1.99 and 1.9 eV, respectively, which are the typical characteristics of semiconductor materials. Varying crystal structures have a significant impact on the properties of materials, with the arrangement of atoms playing a crucial role in determining the crystal structures and influencing charge transfer and electron transitions in response to changing chemical environments.^[^ ^52^, ^58^ ^]^ Compared with medium‐entropy perovskites, high‐entropy perovskites exhibit greater internal chemical disorder. This increased disorder, along with a higher concentration of defects and vacancies, promotes the generation of free electrons and reduces the energy band.^[^ ^18^ ^]^

The density of states (DOS) curve and charge‐density distribution diagram indicate a strong exchange between the Mn─O bond, confirming the presence of double exchange between Mn─O─Mn bonds. The subtle differences between the Mn and O atoms at various sites suggest distortions in the octahedral structure, as shown in Figure 6c,d and Figure S13 (Supporting Information). In addition, the existence of a large number of oxygen vacancies in the sample was ignored during the first‐principles calculations.^[^ ^59^ ^]^ To increase the reliability of the data, UV–vis spectrum measurements and related formulas were used to determine the experimental E_g_ values, as shown in Figure 6e,f.

The bandgap of the specimens was further studied using the *Tauc* plot method based on the formula proposed by *Tauc* et al. The specific expression is as follows:^[^ ^60^, ^61^ ^]^

(9) $${\left(\alpha h\nu \right)}^{1/n}=\;B\left(h\nu -E_{g}\right)$$

where *α* is the absorption coefficient, *hν* is the photon energy, *h* represents *Planck*’s constant (*h* ≈4.13567 × 10‐15 J·s), ν denotes the incident photon frequency (v=c/λ, c is the speed of light (*c*≈3 × 10^8^ m s^−1^), *λ* is the wavelength of the incident light), and *B* is the proportionality constant. The experimentally determined *E_g_* values are slightly lower than the calculated values, indicating that oxygen vacancies play an important role in reducing the bandgap width. In high‐entropy systems, the large number of tailored defects (such as oxygen vacancies and cation disorder) generates a continuous set of intermediate states within the bandgap. These states effectively reduce the bandgap, as confirmed by our UV–vis Tauc plots and DFT calculations. High‐entropy materials exhibit elevated defect concentrations, with defects, particularly oxygen vacancies, serving as sources of electrons. These vacancies provide additional electronic states that facilitate the formation of free carriers. Consequently, electrons are released into the conduction band, thereby enhancing the conductivity of the material. The significant presence of oxygen vacancies can further promote electron generation through mechanisms such as electron transfer or the spin‐polarization effect, thereby improving the electrical conductivity of the materials. In materials characterized by high defect density, defects may engage in nonradiative recombination with conduction‐band electrons. This mechanism does not entirely deplete electrons; instead, it allows them to remain in an excited state within the material for an extended period, thereby contributing to the overall electrical conductivity. Although some recombination occurs, the net effect is an increase in carrier concentration and mobility, resulting in enhanced dielectric loss. Overall, high‐entropy perovskites demonstrate superior absorption attenuation compared to the corresponding terpolymer and medium‐entropy perovskites. The combination of diverse elements and structures in perovskites, coupled with a high‐entropy design, offers exciting potential for novel wave‐absorbing materials, opening new avenues for further exploration and development.

## 3 Conclusion

A series of medium‐ and high‐entropy perovskites with magnetoelectric coordination was successfully synthesized. High‐entropy engineering at the A‐site induces abundant octahedral distortions, oxygen vacancies, and strong interactions between the transition‐metal ions in the high‐entropy (La_0.2_Ba_0.2_Sr_0.2_Ca_0.2_Na_0.2_)MnO_3_ composite. These features render it superior to other polyphase composites and high‐entropy‐absorbing materials. The presence of heterovalent ions at the A‐sites and oxygen vacancies promotes electronic transitions, allowing electrons to hop between cations of different valence states via the oxygen 2p orbital. This facilitates double exchange interactions between Mn^2+^O^−^−Mn^3+^ bonds, resulting in the coupling of magnetic interactions and electron transport, which imparts soft magnetic properties to the material. Therefore, the high‐entropy samples exhibited a minimum reflection loss (RL_min_) of −60.86 dB at a thickness of 1.0 mm and an effective absorption bandwidth of 3.26 GHz, whereas the medium‐entropy ceramics showed RL_min_ values of −17.93 dB at 8.5 mm and −44.59 dB at 8.8 mm. This study offers innovative perspectives in the field of high‐entropy engineering for producing electromagnetic‐wave absorbers.

## 4 Experimental Section

### Raw Materials

Lanthanum nitrate hexahydrate (La(NO_3_)_3_·6H_2_O, AR, 99.99%, Beijing Yinokai Technology Co., LTD., China), Barium chloride monohydrate (BaCl_2_·H_2_O, AR, 99.5%, Tianjin Fengboat Chemical reagent Technology Co., LTD., China), strontium chloride hexahydrate (SrCl_2_·6H_2_O, (AR, 99.5%, Beijing Yinokai Technology Co., LTD., China), calcium chloride (CaCl_2_, AR, 96%, Tianjin Kemi Ou Chemical reagent Technology Co., LTD., China), anhydrous sodium nitrate (NaNO_3_, AR, 99%, Tianjin Fengboat Chemical reagent Technology Co., LTD., China), manganese chloride tetrahydrate (MnCl_2_·4H_2_O, AR, 99%, Tianjin Kemi Ou Chemical Reagent Co., LTD., China), citric acid (C_6_H_8_O_7_·H_2_O, AR, 99%, Beijing Yinokai Technology Co., LTD., China), sodium hydroxide (NaOH, analytical reagent grade, 96%, Tianjin Huadong Reagent Factory, China), anhydrous sodium carbonate (Na_2_CO_3_, analytical reagent grade, 99.8%, Tianjin Fengshan Chemical Reagent Technology Co., Ltd., China), and ethylene glycol ((CH_2_OH)_2_, AR, 99%, Beijing Yinokai Technology Co., LTD, China) were used as raw materials to synthesize high‐entropy ceramics. All the chemical reagents were commercially available.

### Preparation of Perovskite Oxides

A schematic of the experimental procedure is shown in Figure 1a. A sol–gel method was used to prepare high‐entropy ceramic precursors. First, selective raw‐metal salts in stoichiometric ratios were simultaneously dissolved in deionized water. The chelating agent, citric acid, and dispersing agent, ethylene glycol, were then added to the solution at a molar ratio of 1:2 to the metal ions. The mixed solution was heated in an oil bath at 80 °C to form a viscous xerogel. A dry gel precursor powder was obtained by drying in a blast oven. Finally, the obtained precursor was finely pulverized and calcinated in Muffle furnace at various temperatures of 1200, 1300, 1400, and 1450 °C with a heating rate of 5 °C min^−1^ to obtain (La_0.2_Ba_0.2_Sr_0.2_Ca_0.2_Na_0.2_)MnO_3_ high‐entropy ceramics.

Medium‐entropy (Ba_1/3_Sr_1/3_Ca_1/3_)MnO_3_ and ternary manganese‐based oxides, AMnO_3_ (*A* = La, Ba, Sr, Ca, and Na), were synthesized using a coprecipitation method. The metal‐ion salts were dissolved in deionized water at the specified molar ratios to create a metal‐cation solution. Subsequently, excess NaOH and Na_2_CO_3_ were weighed and dissolved in distilled water to obtain the precipitant solution. The metal‐cation solution was then combined with the precipitant solution, followed by static settling, filtration, drying, and ultimately calcination in a muffle furnace at a heating rate of 5 °C min^−1^. All the chemicals used in this study were commercially sourced.

### Characterization

X‐ray diffraction (XRD, PANalytical, Netherlands) using Cu K*α* radiation was employed for phase and crystal structure analysis of powders at room temperature, with a step size 0.013°. The crystal parameters of the synthesized powders were analyzed by Rietveld refinement (FullProf) of the XRD patterns. The medium‐entropy sample (Ba_1/3_Sr_1/3_Ca_1/3_)MnO_3_ was subjected to neutron‐diffraction measurements using a multiphysics instrument with the approval of the China Neutron Source Project. Fine‐tuning of the crystal structure was based on the Rietveld method and was accomplished using the FullProf software. Fine‐tuning of the PDF structure was performed using PDFgui software. The morphologies and elemental distributions of the obtained samples were characterized using SEM (JSM‐IT800, Japan) coupled with EDS. TEM specimens were prepared via micro/nano processing (lift‐out) of the powder using a focused ion beam (Helios NanoLab600i, FEI, USA). SAED measurements were performed using TEM (Talos F200X, FEI, USA) equipped with an energy‐dispersive X‐ray detector (5030, Bruker, USA). HAADF‐STEM (JEM‐ARM300F2, JEOL, Japan) was used to characterize the structure and elemental distribution of (La_0.2_Ba_0.2_Sr_0.2_Ca_0.2_Na_0.2_)MnO_3_ at the atomic scale. XPS was used to determine the composition and chemical states of elements on the surface. The internal oxygen vacancies of the material were characterized using EPR (EMXplus, Bruker, Germany). The magnetic properties of the obtained samples were characterized using a vibrating sample magnetometer (7404, Lakeshore, USA). The high‐entropy powder and paraffin wax were melted and mixed in a 9:1 mass ratio, and then pressed into a ring with outer and inner diameters of 7.00 and 3.04 mm, respectively. The dielectric and magnetic parameters of the samples were measured at frequencies of 2–18 GHz using a vector network analysis instrument (E5080B, Keysight, China).

The electronic properties were calculated based on density functional theory using the Vienna ab initio simulation package.^[^ ^62^ ^]^ The exchange and correlation effects were included using the Heyd—Scuseria–Ernzerhof (HSE06) hybrid functional, which combines short‐range exact exchange with long‐range PBE exchange and correlation.^[^ ^63^ ^]^ The interaction between the atomic core and valence electrons was described using pseudopotentials based on the projector‐augmented plane‐wave method.^[^ ^64^ ^]^ The wave functions for the primitive cell were expanded using plane waves up to an energy cutoff of 550 eV. Integration over the Brillouin zone was sampled with a k‐point grid of 4 × 4 × 4 generated automatically using the Monkhorst–Pack approach.^[^ ^65^ ^]^ The total energy was calculated with high precision and converged to 10^−7^ eV atom^−1^, and the structure relaxation was stopped when the residual forces were less than 10^−4^ eV atom^−1^.

## Conflict of Interest

The authors declare no conflict of interest.

## Supporting information



Supporting Information

## Data Availability

The data that support the findings of this study are available from the corresponding author upon reasonable request.

## References

[1] K. Tang , F. Long , F. Zhang , H. Yin , J. Zhao , M. Xie , Y. An , W. Yang , B. Chi , Nanomaterials 2025, 15, 268. 39997831 10.3390/nano15040268 PMC11858011

[2] Z. Hou , X. Gao , J. Zhang , G. Wang , Carbon N Y 2024, 222, 118935.

[3] B. Hao , Y. Zhang , H. Si , Z. Jiang , C. Li , Y. Zhang , J. Zhang , C. Gong , Adv. Funct. Mater. 2025, 35, 2423897.

[4] Y. Zhu , X. Liu , M. A. Buckingham , P. Acharyya , E. Guilmeau , B. L. Mehdi , D. J. Lewis , R. Freer , J. Eur. Ceram. Soc. 2024, 44, 4666.

[5] X. Yang , A. J. Fernandez‐Carrion , X. Geng , X. Kuang , Pro. Solid State Chem. 2024, 74, 100459.

[6] Y. Liu , H. Dai , Y. Du , J. Deng , L. Zhang , Z. Zhao , Appl. Catal. B. 2012, 119, 20.

[7] Y. Syono , S. Akimoto , K. Kohn , J. Physical Soc. Japan 1969, 26, 993.

[8] Y. Liu , Z. Liu , Z. Li , S. Qin , X. Ye , X. Shen , B. Zhou , G. Zhou , S. Agrestini , M. Valvidares , H. B. Vasili , Z. Hu , Y. Long , Phys. Rev. B. 2020, 101, 144421.

[9] F. Zhao , Z. Yue , Z. Gui , L. I. Longtu , Jpn. J. Appl. Phys. 2005, 44, 8066.

[10] T. Murata , T. Terai , T. Fukuda , T. Kakeshita , Mater. Sci. Forum 2006, 512, 183.

[11] D. Bérardan , S. Franger , D. Dragoe , A. K. Meena , N. Dragoe , Phys. Status Solidi – R 2016, 10, 328.

[12] S. Zhou , Y. Pu , Q. Zhang , R. Shi , X. Guo , W. Wang , J. Ji , T. Wei , T. Ouyang , Ceram. Int. 2020, 46, 7430.

[13] Q. Pang , Y. Chen , Z. Wang , B. Zhou , X. Li , C. Mu , G. Gu , G. Wang , J. Adv. Ceram. 2024, 13, 1382.

[14] W.‐L. Hsu , C.‐W. Tsai , A.‐C. Yeh , J.‐W. Yeh , Nat. Rev. Chem. 2024, 8, 471. 38698142 10.1038/s41570-024-00602-5

[15] Y. Li , Y. Qing , Y. Zhang , H. Xu , J. Adv. Ceram. 2023, 12, 1946.

[16] Y. Ding , K. Ren , C. Chen , L. Huan , R. Gao , X. Deng , G. Chen , W. Cai , C. Fu , Z. Wang , X. Lei , Process Appl. Ceram. 2024, 18, 1.

[17] L. Zhang , J. Jia , J. Yan , Small 2024, 20, 2309586. 10.1002/smll.202309586 38348913

[18] B. Zhao , Z. Yan , Y. Du , L. Rao , G. Chen , Y. Wu , L. Yang , J. Zhang , L. Wu , D. W. Zhang , R. Che , Adv. Mater. 2023, 35, 2210243. 10.1002/adma.202210243 36606342

[19] J. Ma , B. Zhao , H. Xiang , F. Z. Dai , Y. Liu , R. Zhang , Y. Zhou , J. Adv. Ceram. 2022, 11, 754.

[20] G. Dai , R. Deng , X. You , T. Zhang , Y. Yu , L. Song , J. Mater. Sci. Technol. 2022, 116, 11.

[21] L. Huang , M. Wang , L. Cheng , S. Pan , Q. Yao , H. Zhou , J. Alloys Compd. 2022, 892, 162167.

[22] X. Li , H. Yu , S. Gao , X. Fan , D. Zhou , W. Ji , Y. Chen , Y. Zhang , H. Ma , X. Jia , Mater. Charact. 2023, 199, 112792.

[23] H. J. Lee , G. Kim , J.‐S. Kang , B. Dabrowski , S. W. Han , S. S. Lee , C. Hwang , M. C. Jung , H. J. Shin , H. G. Lee , J.‐Y. Kim , B. I. Min , J. Appl. Phys. 2007, 101, 09G523.

[24] M. Li , Q. Zhi , J. Li , C. Wu , X. Jiang , Z. Min , R. Zhang , H. Wang , H. Wang , B. Fan , J. Mater. 2024, 10, 1176.

[25] X. Guo , J. Kang , R. Gu , J. Wang , L. Jin , X. Wei , Ceram. Int. 2024, 50, 14232.

[26] S. B. Mary , K. S. Mohan , M. M. Krishnan , J. Mater. Sci.‐Mater. E. 2024, 35, 600.

[27] M. Molinari , D. A. Tompsett , S. C. Parker , F. Azough , R. Freer , J. Mater. Chem. A. 2014, 2, 14109.

[28] E. A. Eno , D. Etiese , K. Pathmanathan , E. C. Agwamba , U. G. Chukwu , T. O. Magu , A. I. Ikeuba , A. S. Adeyinka , H. Louis , Chem. Phys. Impact 2023, 7, 100321.

[29] K. Yang , D. I. Khomskii , H. Wu , Phys. Rev. B. 2018, 98, 085105.

[30] R. Witte , A. Sarkar , R. Kruk , B. Eggert , R. A. Brand , H. Wende , H. Hahn , Phys. Rev. Mater. 2019, 3, 034406, 10.1103/PhysRevMaterials.3.034406.

[31] P. Zhang , L. Gong , Z. Lou , J. Xu , S. Cao , J. Zhu , H. Yan , F. Gao , J. Alloys Compd. 2022, 898, 162858.

[32] S. Jiang , T. Hu , J. Gild , N. Zhou , J. Nie , M. Qin , T. Harrington , K. Vecchio , J. Luo , Scr. Mater. 2018, 142, 116.

[33] J. Shi , X. Liu , F. Zhu , W. Tian , Y. Xia , T. Li , R. Rao , T. Zhang , L. Liu , J. Mater. 2022, 8, 719.

[34] S. Huang , L. Deng , K. Zhou , Z. Hu , S. Sun , Y. Ma , P. Xiao , J. Magn. Magn. Mater. 2012, 324, 3149.

[35] J. Liu , G. Shao , D. Liu , K. Chen , K. Wang , B. Ma , K. Ren , Y. Wang , Mater. Today. Adv. 2020, 8, 100114.

[36] A. Sarkar , R. Djenadic , D. Wang , C. Hein , R. Kautenburger , O. Clemens , H. Hahn , J. Eur. Ceram. Soc. 2018, 38, 2318.

[37] Y. Zhang , J. Feng , F. Zhang , M. Ma , Z. Liu , J. Amer. Ceram. Soc. 2024, 107, 3497.

[38] K. Zhao , W. Zhong , M. Sun , L. Chen , D. Liu , J. Liu , Adv. Eng. Mater. 2024, 26, 2400695.

[39] M. C. Biesinger , B. P. Payne , A. P. Grosvenor , L. W. M. Lau , A. R. Gerson , R. St. C. Smart , Appl. Surf. Sci. 2011, 257, 2717.

[40] X. Sun , X. Zeng , X. He , W. Fang , X. Du , W. Li , L. Zhao , H. Chen , J. Alloys Compd. 2022, 925, 166560.

[41] M. Li , J. P. Hodges , M. Retuerto , Z. Deng , P. W. Stephens , M. C. Croft , X. Deng , G. Kotliar , J. Sánchez‐Benítez , D. Walker , M. Greenblatt , Chem. Mater. 2016, 28, 3148.

[42] J. Hejtmánek , K. Knížek , M. Maryško , Z. Jirák , D. Sedmidubsk , O. Jankovský , Š. Huber , P. Masschelein , B. Lenoir , J. Appl. Phys. 2012, 111, 07D715.

[43] R. Witte , A. Sarkar , L. Velasco , R. Kruk , R. A. Brand , B. Eggert , K. Ollefs , E. Weschke , H. Wende , H. Hahn , J. Appl. Phys. 2020, 127, 185109.

[44] X. Zeng , X. Cheng , R. Yu , G. D. Stucky , Carbon N Y 2020, 168, 606, 10.1016/j.carbon.2020.07.028.

[45] Y. Li , Y. Qing , Y. Zhou , B. Zhao , Q. Zhi , B. Fan , R. Zhang , Compos. B‐ Eng. 2021, 213, 108731.

[46] D. Tan , Q. Wang , M. Li , L. Song , F. Zhang , Z. Min , H. Wang , Y. Zhu , R. Zhang , D. Lan , B. Fan , Chem. Eng. J. 2024, 492, 152245.

[47] R. Peymanfar , H. Dogari , E. Selseleh‐Zakerin , M. H. Hedayatzadeh , S. Daneshvar , N. Amiri‐Ramsheh , H. Ghafuri , A. Mirkhan , G. Ji , B. Aslibeiki , Front Mater. 2023, 10, 1133287.

[48] J. Shen , M. Zhang , S. Lin , W. Song , H. Liu , Q. Liu , X. Zhu , Y. Sun , J. Appl. Phys. 2023, 133,235101.

[49] Y. Li , Y. Qing , Y. Cao , F. Luo , H. Wu , Small 2023, 19, 2302769. 10.1002/smll.202302769 37292045

[50] X. B. Xie , B. Wang , Y. Wang , C. Ni , X. Sun , W. Du , Chem. Eng. J. 2022, 428, 131160.

[51] W. Zhang , F. Z. Dai , H. Xiang , B. Zhao , X. Wang , N. Ni , R. Karre , S. Wu , Y. Zhou , J. Adv. Ceram. 2021, 10, 1299.

[52] B. Zhao , Y. Du , Z. Yan , L. Rao , G. Chen , M. Yuan , L. Yang , J. Zhang , R. Che , Adv. Funct. Mater. 2023, 33, 2209924.

[53] W. Wang , G. Sun , X. Sun , Z. Zhang , J. Zhang , Y. Liang , J. Bi , Mater. Res. Bull 2023, 163, 112212.

[54] Z. Yan , D. Li , X. Zhang , Q. Men , B. Fan , L. Guan , X. Guo , R. Zhang , B. Zhao , Ceram. Int. 2022, 48, 36871.

[55] A. Sarkar , R. Kruk , H. Hahn , Dalton Trans. 2021, 50, 1973. 33443275 10.1039/d0dt04154h

[56] H. Wu , J. Liu , H. Liang , D. Zang , Chem. Eng. J. 2020, 393, 124743.

[57] L. Qiao , J. Bi , G. Liang , Y. Yang , H. Wang , S. Wang , J. Adv. Ceram. 2023, 12, 1902.

[58] Y. Zhou , B. Zhao , H. Chen , H. Xiang , F. Z. Dai , S. Wu , W. Xu , J. Mater. Sci. Technol. 2021, 74, 105.

[59] X. Liu , Y. Xing , K. Xu , H. Zhang , M. Gong , Q. Jia , S. Zhang , W. Lei , Small 2022, 18, 2200524. 10.1002/smll.202200524 35362260

[60] E. Pachoud , J. Cumby , C. T. Lithgow , J. P. Attfield , J. Am. Chem. Soc. 2018, 140, 636. 29258310 10.1021/jacs.7b09441

[61] L. L. Xie , T. F. Shi , J. C. Lin , X. K. Zhang , X. K. Zhong , K. K. Liu , B. K. Dong , C. Yang , X. L. Wang , T. J. Xiong , W. S. Yan , J. P. Xu , H. C. Chen , W. Yin , M. Li , P. Tong , W. H. Song , Y. P. Sun , J. Mater. Sci. Technol. 2023, 146, 80.

[62] G. Kresse , J. Furthmüller , Phys. Rev. B. 1996, 54, 11169. 10.1103/physrevb.54.11169 9984901

[63] J. Heyd , G. E. Scuseria , M. Ernzerhof , J. Chem. Phys. 2003, 118, 8207.

[64] G. Kresse , D. Joubert , Phys. Rev. B. 1999, 59, 1758.

[65] H. J. Monkhorst , J. D. Pack , Phys. Rev. B. 1976, 13, 5188.

