# Supplementary material for: Rational A‐Site Entropy Engineering in Perovskites: Dual‐Exchange Enhanced Magnetoelectric Coupling for Ultra‐Efficient Microwave Absorption
Source: Adv Sci (Weinh). 2025 Oct 17;13(1):e16938. doi: 10.1002/advs.202516938 (PMC12766988; doi:10.1002/advs.202516938)
Supplement: Supplementary file 1 — Supporting Information [file ADVS-13-e16938-s001.docx]

**Rational A-Site Entropy Engineering in Perovskites: Dual-Exchange Enhanced Magnetoelectric Coupling for Ultra-Efficient Microwave Absorption**

Mengru Li*^a^*, Kaiyue Zhao*^a,b^*, Bingbing Fan*^a, c, d*^*, Yang Li*^e^*, Dalong Tan*^a^*, Hailong Wang*^a^*, Qilong Gao*^a,b^*, Wei Li*^a^*, Hongsong Zhang*^f^*, Yanqiu Zhu*^d^*, Rui Zhang*^a, c, g*^*

*^a^* School of Materials Science and Engineering, Zhengzhou University, Zhengzhou 450001, China

*^b^* School of Physics, Zhengzhou University, Zhengzhou 450001, China

*^c^* Henan Academy of Sciences, Zhengzhou 450046, China

*^d^* Department of Engineering, Faculty of Environment, Science and Economy, University of Exeter, Exeter EX4 4QF, United Kingdom

*^e^* State Key Laboratory of Structural Analysis Optimization and CAE Software for Industrial Equipment, National Engineering Research Center for Advanced Polymer Processing Technology, Zhengzhou University, Zhengzhou, 450002, China

*^f^* Mechanical Engineering, Henan University of Engineering, 451151, China

*^g^* School of Materials Science and Engineering, Luoyang Institute of Technology, Luoyang 471000, China

*Corresponding author.

E-mail: fanbingbing@zzu.edu.cn (B. Fan) zhangray@zzu.edu.cn (R. Zhang)

**Table S1**. The radius of cations and oxygen ion and the *t* value of materials

| ions | Mn^2+^ | Mn^3+^ | Mn^4+^ | La^3+^ | Ba^2+^ | Sr^2+^ | Ca^2+^ | Na^+^ | O^2-^ |
| --- | --- | --- | --- | --- | --- | --- | --- | --- | --- |
| Radius (Å) | 0.830 | 0.645 | 0.530 | 1.032 | 1.35 | 1.18 | 1.00 | 1.020 | 1.400 |
| Goldschmidt (*t*)  0.77-1.10 | (Ba_1/3_Sr_1/3_Ca_1/3_)MnO_3_ | | | | | | | | 0.817-0.944 |
|  | (La_0.25_Ba_0.25_Sr_0.25_Ca_0.25_)MnO_3_ | | | | | | | | 0.804-0.929 |
|  | (La_0.2_Ba_0.2_Sr_0.2_Ca_0.2_Na_0.2_)MnO_3_ | | | | | | | | 0.797-0.921 |


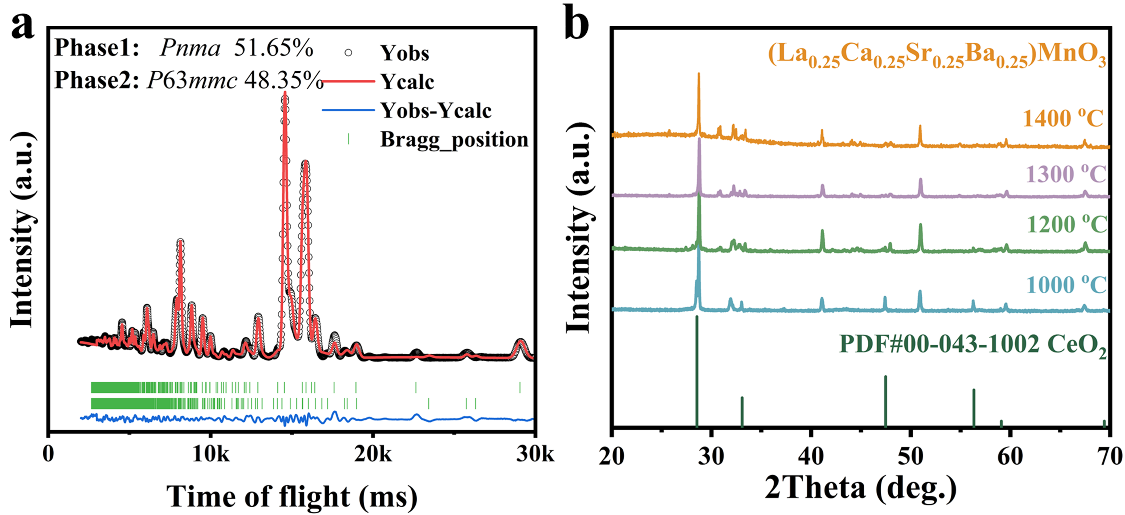


**Figure S1** (a) Neutron diffraction pattern of medium-entropy (Ba_1/3_Sr_1/3_Ca_1/3_)MnO_3_ and (b) XRD patterns of (La_0.25_Ba_0.25_Sr_0.25_Ca_0.25_)MnO_3_


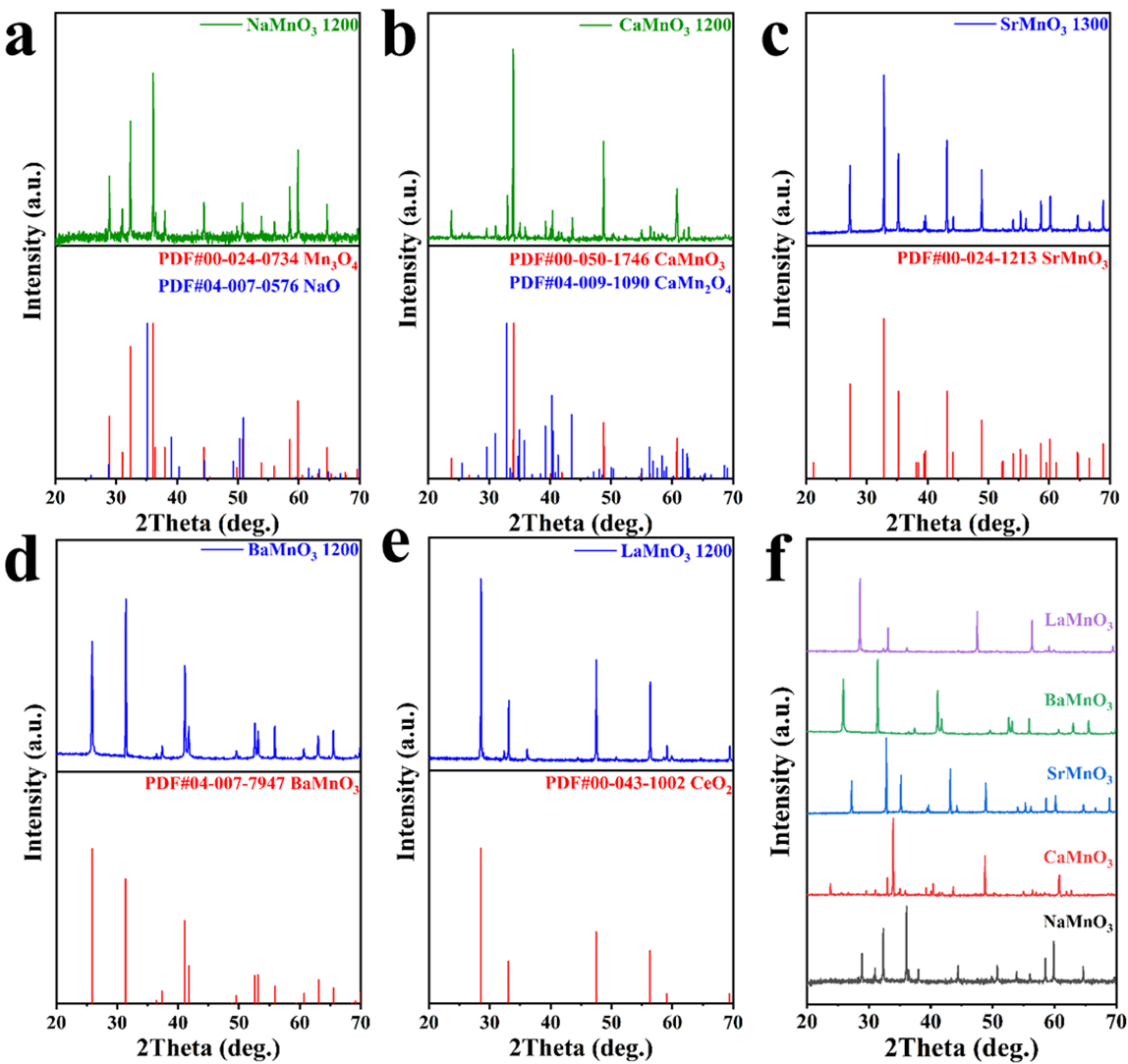


**Figure S2** XRD patterns of Mn matrix ternary perovskite (a) NaMnO_3_, (b) CaMnO_3_, (c) SrMnO_3_, (d) BaMnO_3_, (e) LaMnO_3_, and (e) their comparison diagram


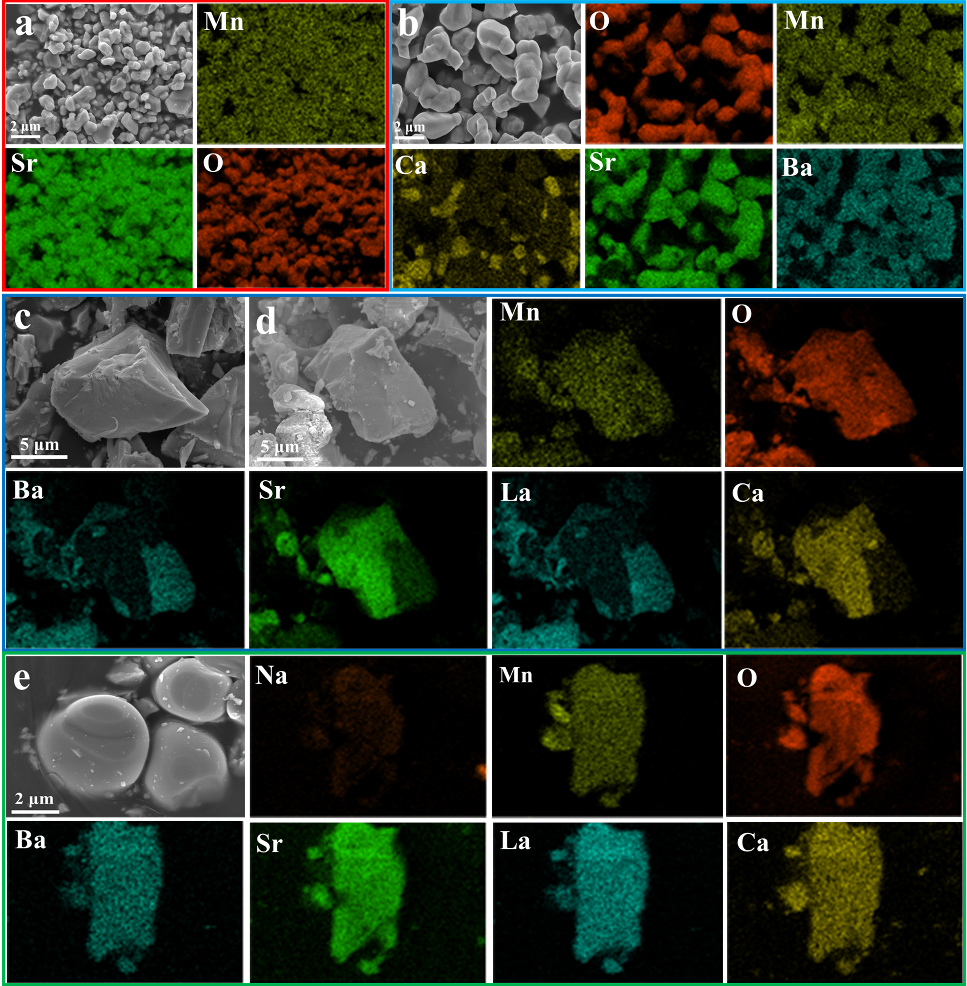


**Figure S3** EM and equipped EDS images of (a) SrMnO_3_, (b) (Ba_1/3_Sr_1/3_Ca_1/3_)MnO_3_, (c) (La_0.25_Ba_0.25_Sr_0.25_Ca_0.25_)MnO_3_, and (d) (La_0.2_Ba_0.2_Sr_0.2_Ca_0.2_Na_0.2_)MnO_3_


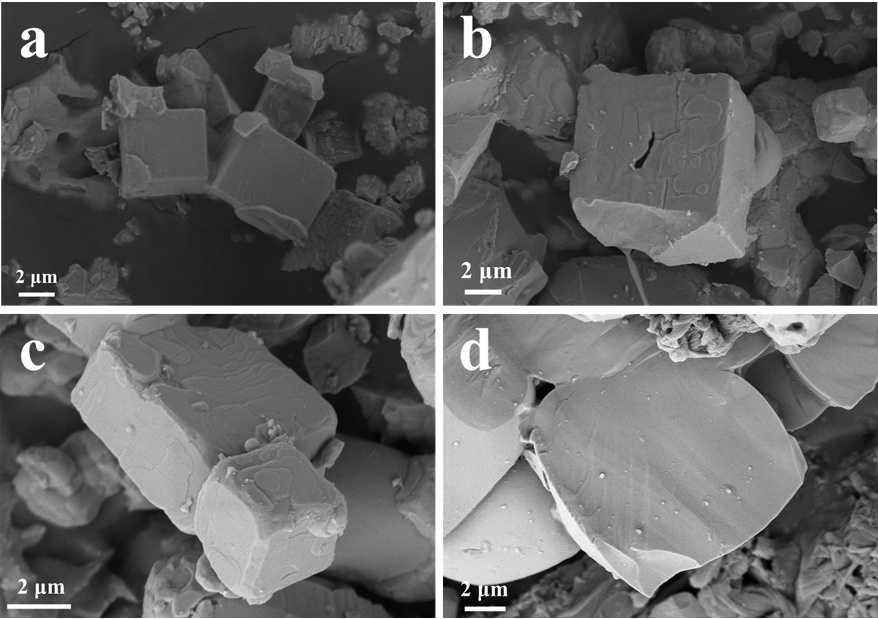


**Figure S4** SEM of (La_0.2_Ba_0.2_Sr_0.2_Ca_0.2_Na_0.2_)MnO_3_ in sintering temperature at (a) 1200 ^o^C, (b) 1300 ^o^C, (c) 1400 ^o^C, (d) 1450 ^o^C


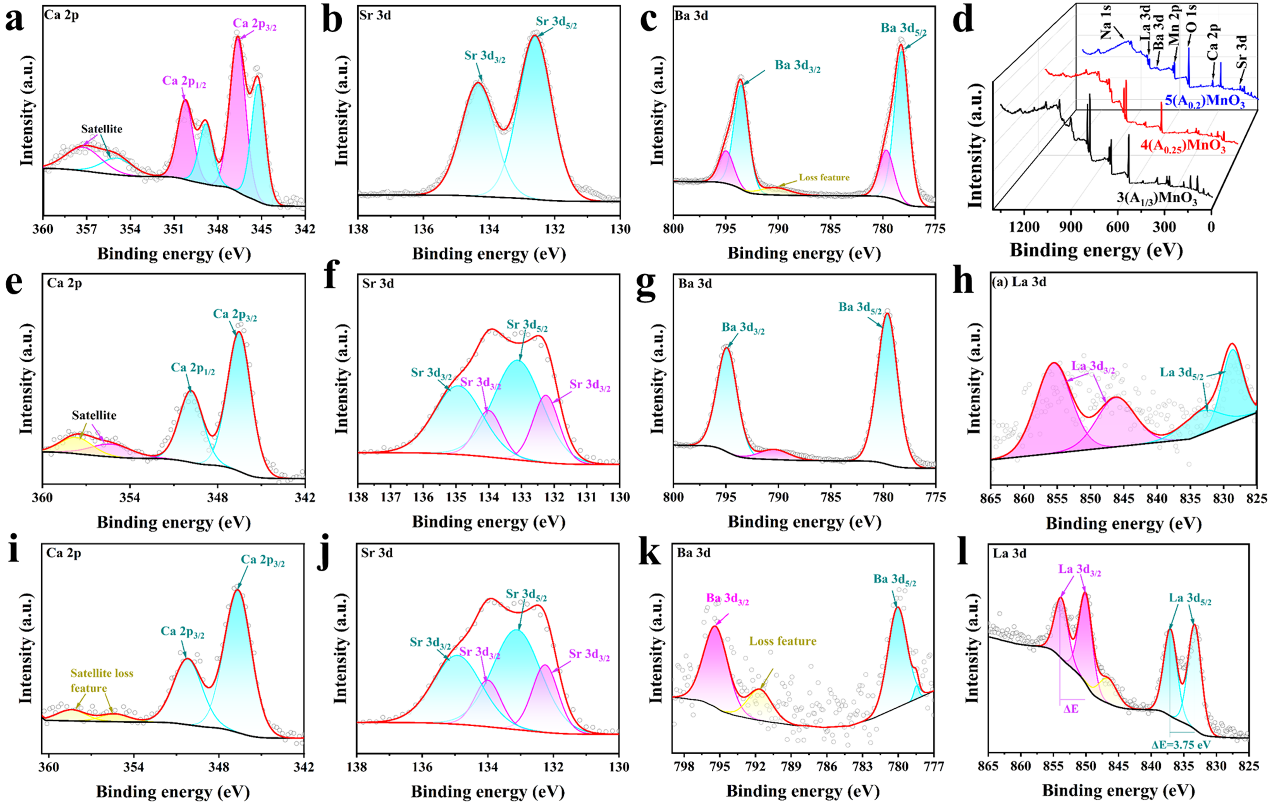


**Figure S5** XPS spectras of (a, e, i) Ca, (b, f, j) Sr, (c, g, k) Ba, (h, l) and (d) survey of (Ba_1/3_Sr_1/3_Ca_1/3_)MnO_3_, (La_0.25_Ba_0.25_Sr_0.25_Ca_0.25_)MnO_3_, and (La_0.2_Ba_0.2_Sr_0.2_Ca_0.2_Na_0.2_)MnO_3_, accordingly.


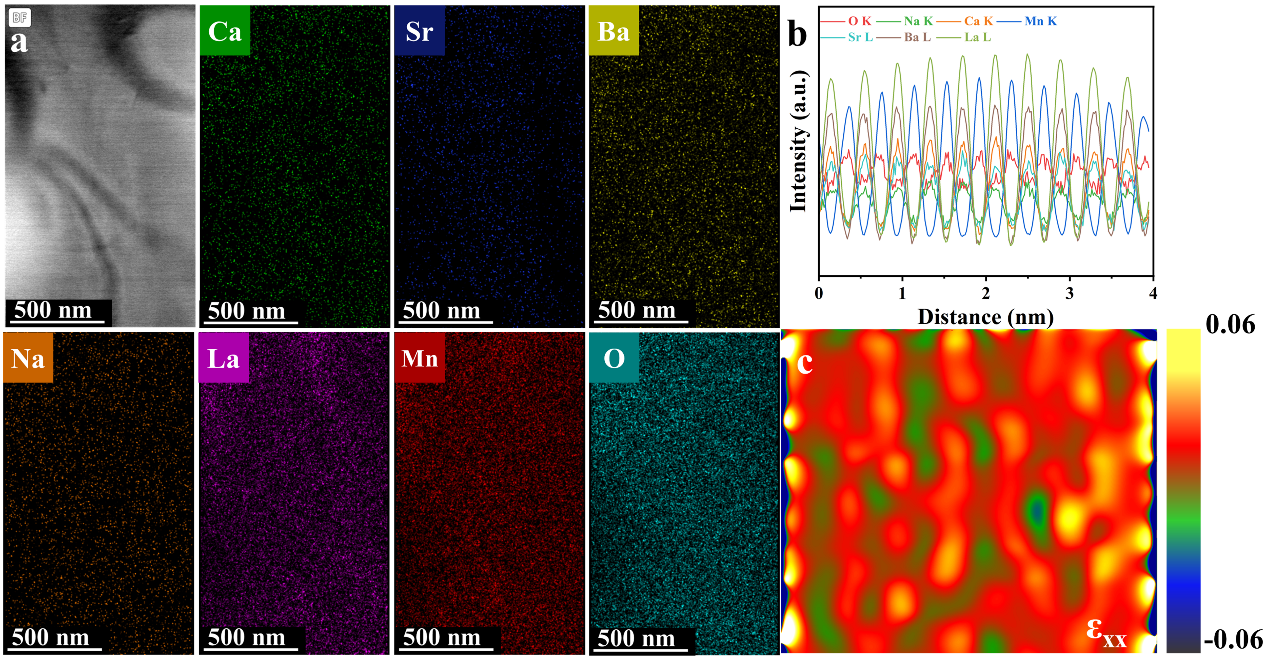


**Figure S6** (a) TEM equipped elemental mapping, (b) the aberration line scan curves of each element in STEM and (c) the corresponding strain field ε_xx_ of STEM image was obtained by GPA method


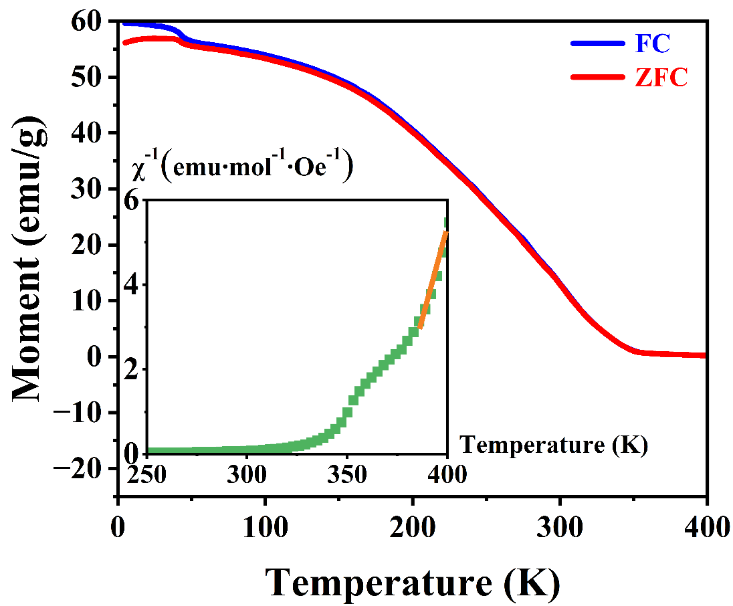


**Figure S7** M-T curve of high-entropy ceramic (La_0.2_Ba_0.2_Sr_0.2_Ca_0.2_Na_0.2_)MnO_3_, illustrated as χ^-1^-T curve with temperature.


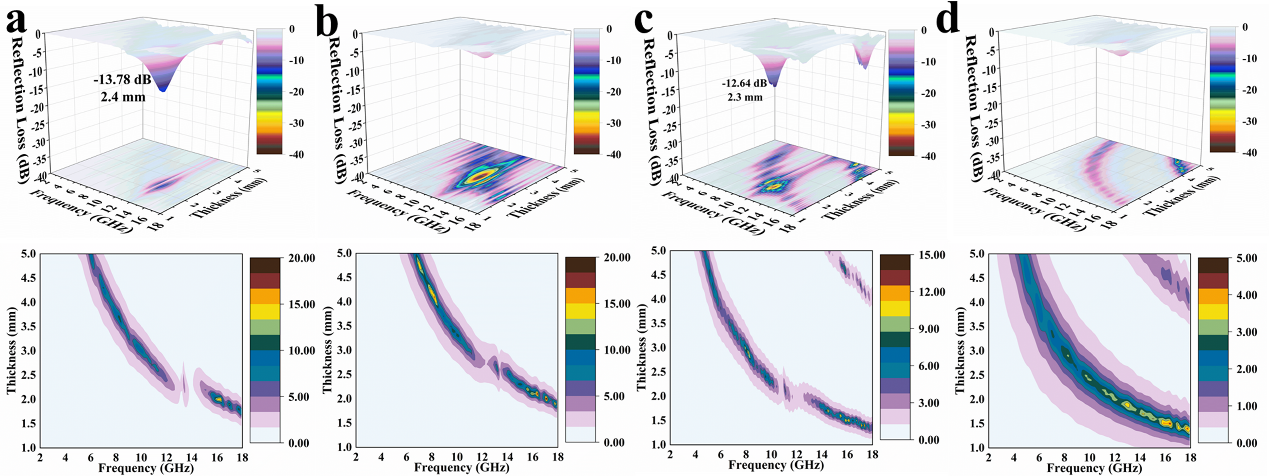


**Figure S8** 3D diagrams of reflection loss and impedance matching of (a) LaMnO_3_, (b) BaMnO_3_, (c) SrMnO_3_ and (d) CaMnO_3_


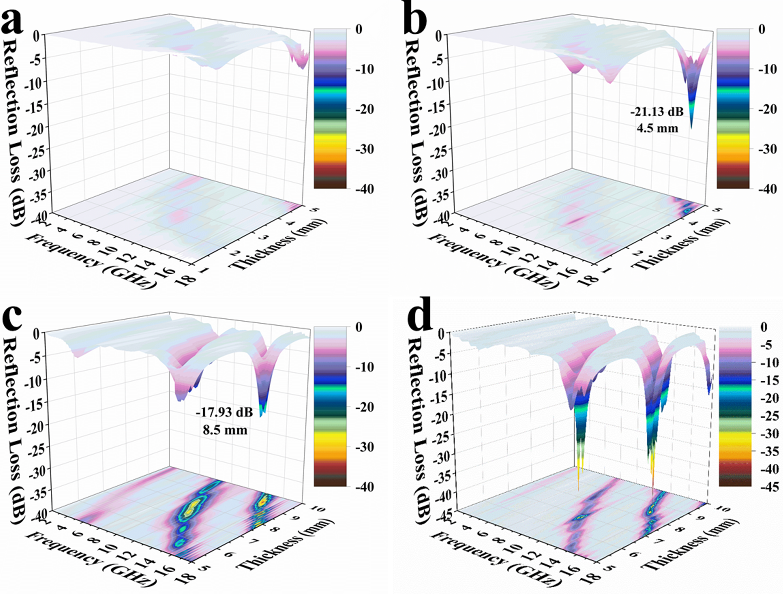


**Figure S9** 3D diagrams of reflection loss of (Ba_1/3_Sr_1/3_Ca_1/3_)MnO_3_ and (La_0.25_Ba_0.25_Sr_0.25_Ca_0.25_)MnO_3_ with thickness (a-b) 1-5 mm and (c-d) 5-10 mm, correspondingly.

**
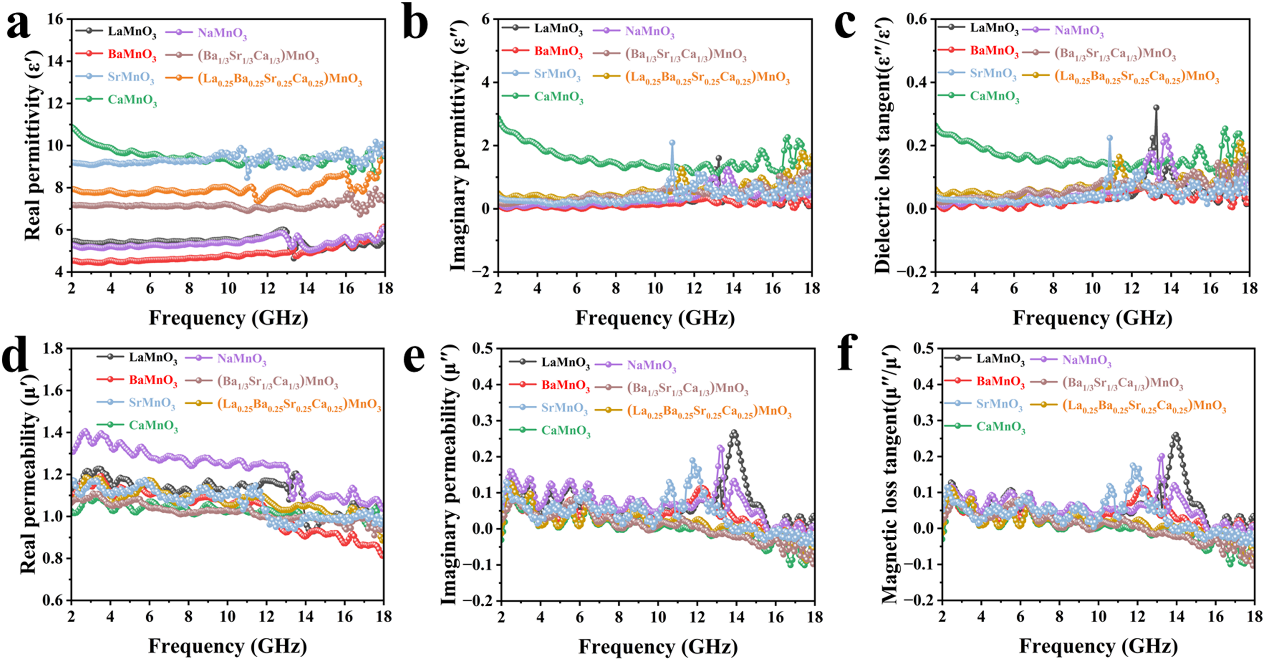
**

**Figure S10** Electromagnetic parameters of Mn-base oxides, (a) Real permittivity (*εʹ*), (b) imaginary permittivity (*ε"*), (c) dielectric loss tangent(*ε"/εʹ*), (d) real permeability (*μʹ*), (e) imaginary permeability (*μ"*), and (f) magnetic loss tangent(*μ"/μʹ*)


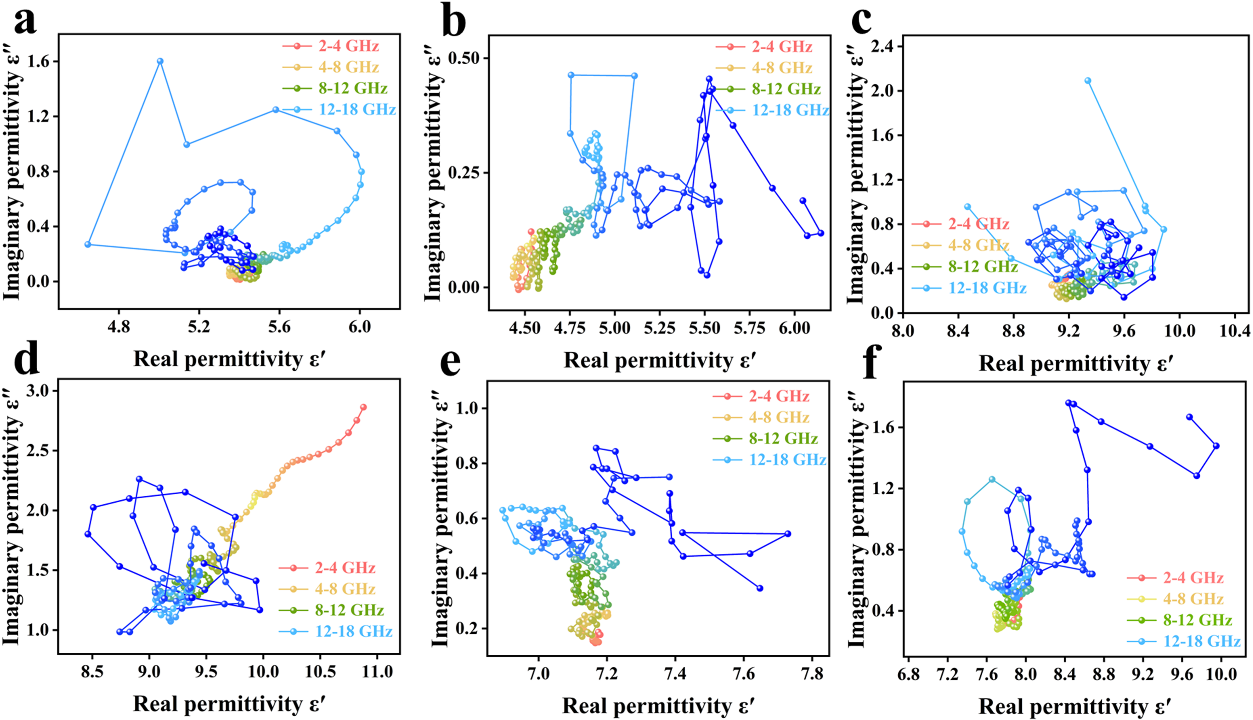


**Figure S11** Cole-Cole circles of (a) LaMnO_3_, (b) BaMnO_3_, (c) SrMnO_3_, (d) CaMnO_3_, (e) (Ba_1/3_Sr_1/3_Ca_1/3_)MnO_3_ and (f) (La_0.25_Ba_0.25_Sr_0.25_Ca_0.25_)MnO_3_


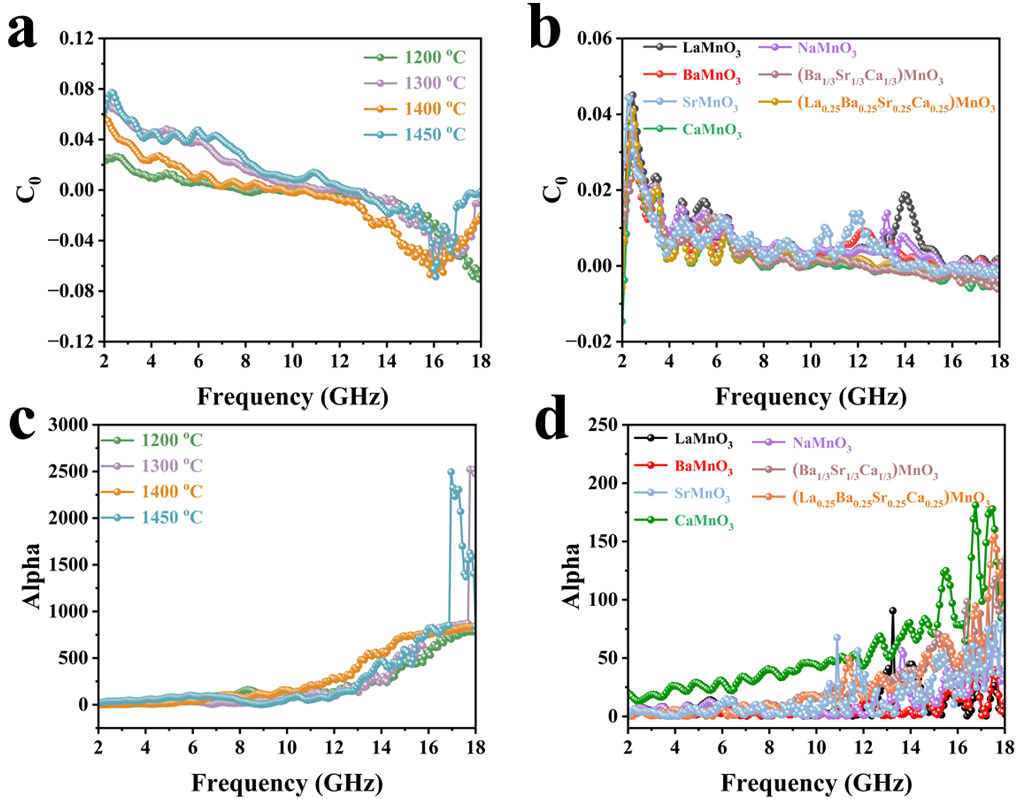


**Figure S12** *C_0_* and attenuation constant (*α*) of (a, c) High-entropy ceramics with various fired temperature and (b, d) Mn-base oxides.


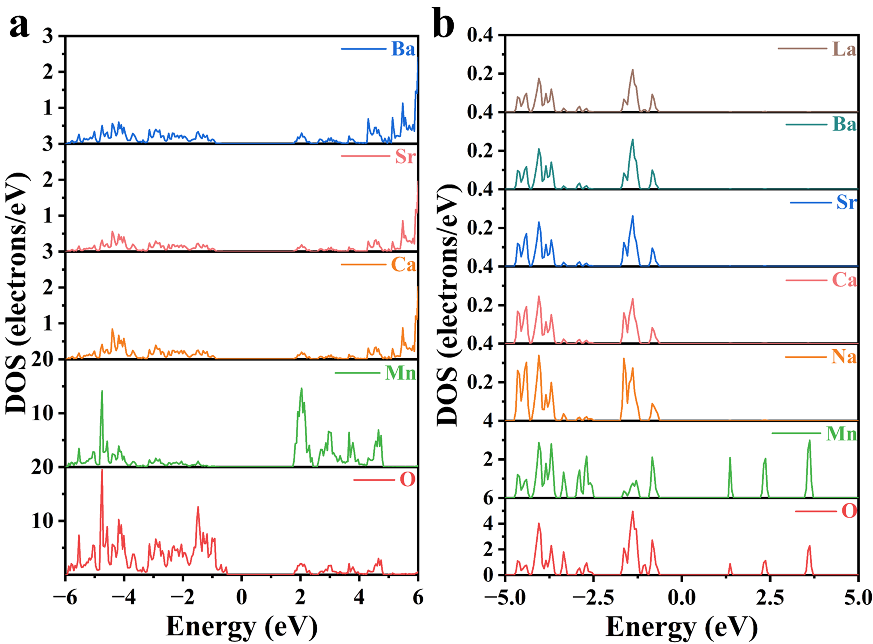


**Figure S13** Density of states plots for (a) medium- and (b) high-entropy.
